# Reactive Cysteines in Proteins are the Dominant Reductants for Platinum(IV) Prodrug Activation in Live Cells

**DOI:** 10.1002/anie.202416396

**Published:** 2025-04-26

**Authors:** Shu Chen, Wang Peng, Houzong Yao, Zhiqin Deng, Zhao Yue, Gongyuan Liu, Jiaqian Xu, Naixin Lin, Weikang Xu, Jianbo Yue, Guangyu Zhu

**Affiliations:** ^1^ Department of Chemistry City University of Hong Kong 83 Tat Chee Ave, Kowloon Tong Hong Kong SAR P.R. China; ^2^ City University of Hong Kong Shenzhen Research Institute Shenzhen P.R. China; ^3^ Department of Biomedical Sciences City University of Hong Kong 83 Tat Chee Ave, Kowloon Tong Hong Kong SAR P.R. China

**Keywords:** Bioluminescence imaging, Live cells, Platinum(IV) prodrug, Platinum(IV) reduction, Prodrug activation

## Abstract

The intracellular reduction of Pt(IV) prodrugs is crucial for their anticancer efficacy. However, the major components responsible for the reduction of Pt(IV) complexes within live cells remain elusive. Herein, we developed an aminoluciferin‐functionalized Pt(IV) complex, **Pt‐Luc**, that can be used as a bioluminescent reporter for real‐time monitoring of Pt(IV) reduction in live cancer cells by capturing immediate bioluminescent signals from the released aminoluciferin. Utilizing this powerful reporter, we found that the reduction of Pt(IV) prodrugs in live cancer cells significantly slows down when cysteine levels are reduced, while the levels of glutathione do not impact the reduction rate. Further investigation reveals that reactive cysteines in proteins, rather than small‐molecule thiols, play a primary role in reducing the Pt(IV) complex. In vivo studies reveal a substantial 63% decrease in bioluminescence from **Pt‐Luc** in thiol‐blocking tumors in mice, reinforcing the pivotal role of reactive cysteines in Pt(IV) reduction. This study provides valuable insights into the activation mechanisms of Pt(IV) prodrugs in live cells and in vivo, enhancing our understanding of prodrug activation beyond buffer systems or fixed cells.

## Graphical abstract

**Pt‐Luc** was developed as a pioneering bioluminescent reporter for real‐time monitoring of Pt(IV) reduction in live cells. Using this advanced reporter, we identified that reactive cysteines in proteins, rather than those in small molecules, predominantly mediate the reduction of Pt(IV) prodrugs. Furthermore, the thiol‐mediated reduction of Pt(IV) complexes was corroborated in vivo.

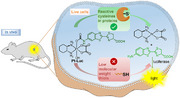

## Introduction

Bioluminescent imaging (BLI) is a powerful tool that facilitates real‐time visualization of biological features within tissues and cells.^[^ ^1^, ^2^, ^3^, ^4^ ^]^ This innovative technology employs luciferase enzymes and the small‐molecule luciferin to produce visible light, thereby enabling a sensitive and noninvasive assessment of physiological functions in tissues and living organisms.^[^ ^5^, ^6^, ^7^, ^8^, ^9^ ^]^ A key advantage of BLI is its ability to image bioluminescent events in real time, enabling immediate capture and visualization of images or videos without significant delays.^[^ ^10^, ^11^, ^12^ ^]^ Furthermore, the intrinsic feature of bioluminescence eliminates the requirement for excitation light,^[^ ^13^ ^]^ allowing BLI to overcome the limitations of in vivo fluorescence imaging, such as high tissue‐derived autofluorescence.^[^ ^14^ ^]^ A cutting‐edge approach in BLI involves caging luciferin by conjugating it with various functional groups, offering a valuable toolkit for investigating diverse biological processes.^[^ ^15^, ^16^, ^17^, ^18^, ^19^ ^]^ The reliability, sensitivity, convenience, and non‐invasiveness of BLI have significantly advanced our understanding of fundamental in vivo biology.

**Scheme 1:**
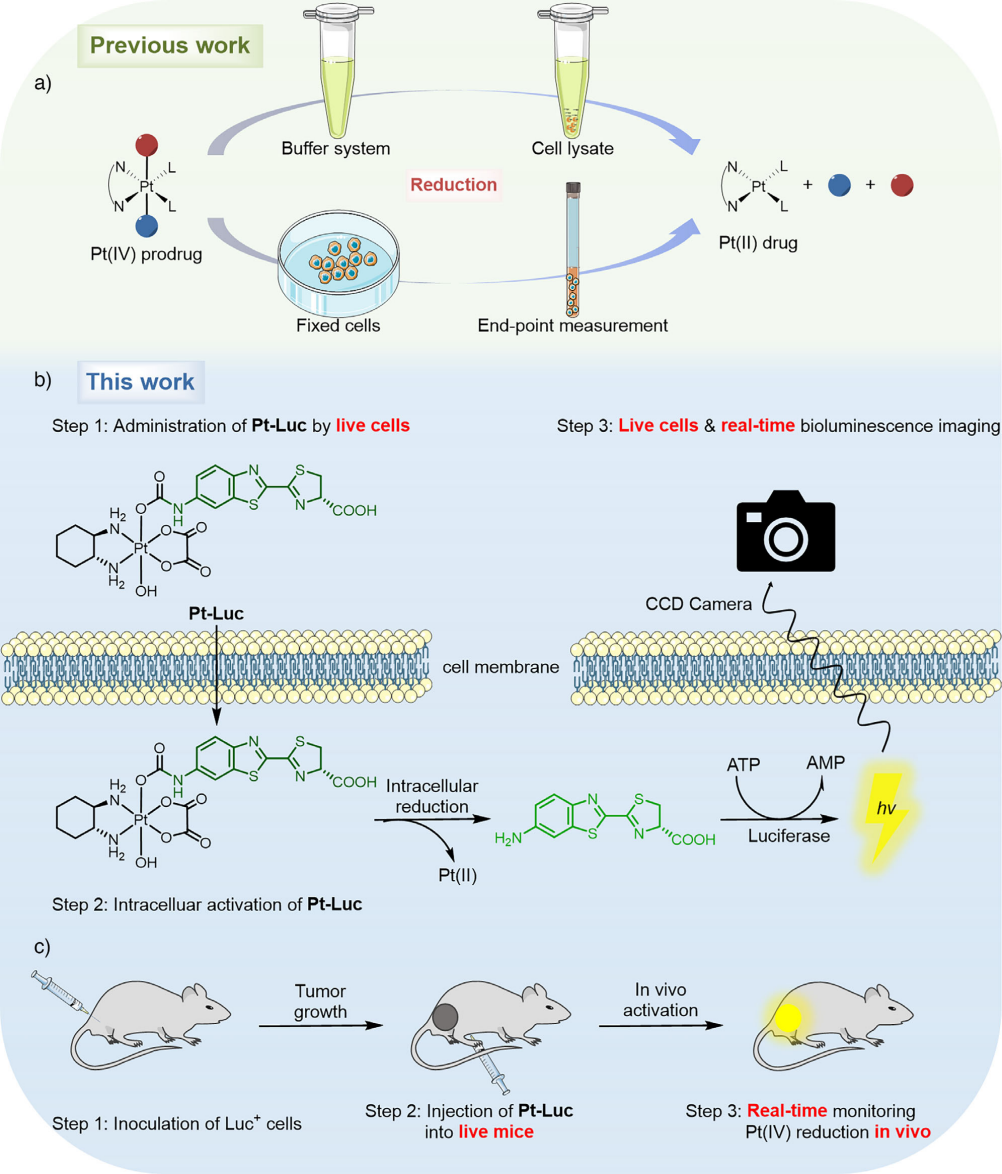
a) A scheme demonstrating that previous investigations of Pt(IV) reduction were limited to buffer systems, cell lysates, fixed cells, or measurement only at the endpoint. b,c) A bioluminescent reporter system that enables real‐time imaging of Pt(IV) reduction in live cells and animal models.

Recently, Pt(IV) prodrugs have demonstrated remarkable progress in reducing side effects, enhancing drug efficiency, and overcoming resistance, positioning them as promising candidates for next‐generation platinum‐based antineoplastics.^[^ ^20^, ^21^, ^22^, ^23^, ^24^, ^25^, ^26^ ^]^ The intracellular reduction of these prodrugs is essential for their anticancer effects, underscoring the importance of comprehending this process.^[^ ^27^, ^28^, ^29^, ^30^, ^31^, ^32^, ^33^ ^]^ Extensive research has been dedicated to exploring the reduction mechanisms of Pt(IV) prodrugs (Scheme 1a). In buffer systems, small molecules such as ascorbate, glutathione (GSH), and cysteine have been posited to play pivotal roles in reducing Pt(IV) complexes.^[^ ^21^, ^31^, ^34^ ^]^ However, cell lysate studies have contradicted these perspectives, demonstrating that high molecular weight (HMW) fractions, rather than low molecular weight (LMW) fractions containing small molecules, are more effective in reducing Pt(IV) carboxylates.^[^ ^35^, ^36^ ^]^ Although various cell‐based studies have been conducted utilizing fluorescent probes and isotopically labeled ligands,^[^ ^37^, ^38^, ^39^ ^]^ the key components responsible for the reduction of Pt(IV) prodrugs in live cells remain elusive. There is a significant gap in real‐time monitoring of Pt(IV) complex reduction in live cells, primarily due to the lack of real‐time monitoring tools. Hence, it is imperative to devise innovative approaches that enable real‐time monitoring of Pt(IV) reduction in live cells, thereby revealing the underlying mechanisms of Pt(IV) prodrug reduction at the cellular level.

In this study, we developed a bioluminescent reporter system that enables the imaging of Pt(IV) reduction in live cells and animal models (Scheme 1b,c). This reporter is elaborately designed by incorporating an amino‐based luciferase substrate onto the axial position of a Pt(IV) prodrug, creating a carbamate‐functionalized Pt(IV) complex that effectively balances long‐term stability and prompt reduction. Furthermore, the system amalgamates the advantages of low background, high sensitivity, real‐time imaging, and non‐invasiveness. We demonstrated the ability of the probe to detect the reduction process of Pt(IV) prodrugs in live cells in real time. Utilizing this potent probe, we found that the depletion of GSH does not significantly impact Pt(IV) reduction in live cells, whereas blocking thiol levels results in a substantial decrease in reduction rate. Further investigation revealed that reactive cysteines in proteins are the dominant agents responsible for the reduction of Pt(IV) prodrugs. In a murine model of lung cancer, in vivo imaging using the probe showed a significant decrease in bioluminescence in thiol‐blocking mice, highlighting the role of thiols in Pt(IV) reduction. This work represents the first successful attempt at monitoring the reduction of Pt(IV) prodrugs in a real‐time, noninvasive, and live‐cell manner, advancing our understanding of the components involved in the process both in live cells and in vivo.

## Results and Discussion

The design of the bioluminescence probe was based on BLI, which uses light‐generating luciferase enzymes along with their appropriate substrates, namely, *D*‐luciferin and aminoluciferin scaffolds.^[^ ^11^, ^15^ ^]^ Prior studies have highlighted the significance of the 6ʹ‐amino group in aminoluciferin for enzyme interactions. Acylation at the 6ʹ‐amino position often hinders luciferase recognition and quenches bioluminescence emission.^[^ ^15^ ^]^ Building on these findings, we proposed that the functionalization of the 6ʹ‐amino group of aminoluciferin with Pt(IV) complexes can facilitate bioluminescence quenching. Upon reduction of the Pt(IV) complex, the caged substrate is cleaved, releasing free aminoluciferin, which is oxidized by luciferase to emit light. The intensity of the emitted light directly correlates with the amount of free aminoluciferin, reflecting the extent of Pt(IV) complex reduction (Scheme 1b,c).

The probe, designated **Pt‐Luc**, was synthesized and fully characterized by ESI‐HRMS, ^1^H, ^13^C, ^195^Pt NMR, and HPLC (Scheme S1 and Figures S1–S9). The hydrolytic stability of **Pt‐Luc** was assessed in a phosphate buffer (50 mM, pH 7.4) using HPLC analysis. After 6 h, **Pt‐Luc** remained intact, with no hydrolyzed ligand, i.e., free aminoluciferin, detected (Figure S10a,b). Additionally, the stability of **Pt‐Luc** was evaluated in a cell‐culture medium, where the probe exhibited resistance to hydrolysis (Figure S10c,d). The reduction reaction was performed in a phosphate buffer (50 mM, pH 7.4) containing sodium ascorbate. The reduction of **Pt‐Luc** was completed after 2 h, with a half‐life (t_1/2_) of 46 min (Figures S10e,f, S11, and S12).

**Figure 1:**
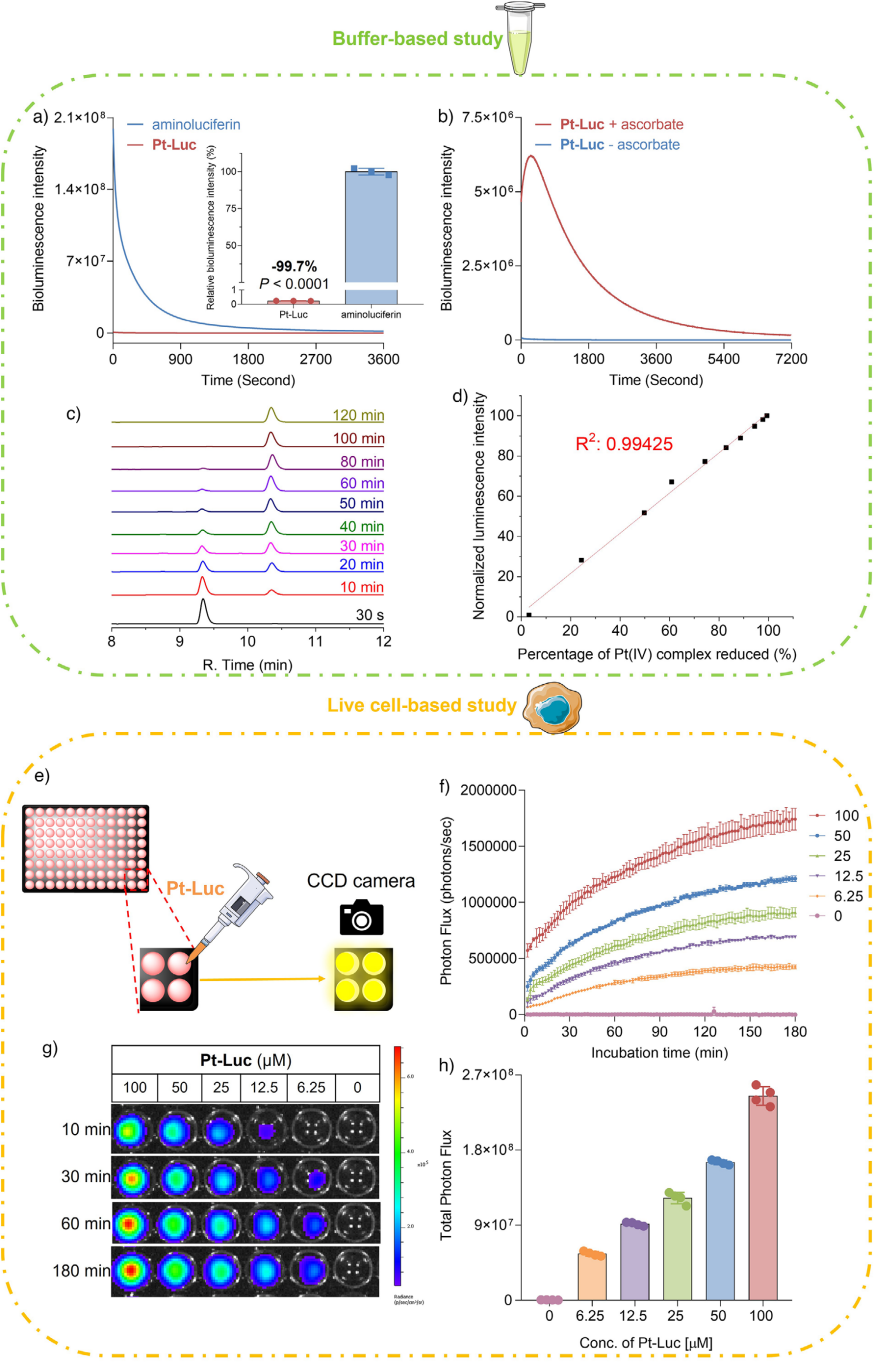
Establishing the linear relationship between the bioluminescence increment and the amount of Pt(IV) complex reduced in buffers. a) Time‐dependent bioluminescent signal generation of **Pt‐Luc** and aminoluciferin in Tris buffer (50 mM pH 7.4) containing luciferase (100 µg mL^−1^, Promega), 10 mM Mg^2+^ (MgCl_2_), 0.1 mM Zn^2+^ (ZnCl_2_), and 2 mM ATP. Bioluminescent signals were measured at 37 °C for 1 h. Inset: integrated and normalized bioluminescent signals from the dynamic curve of (a). Total photon flux was integrated over 1 h. b) Time‐dependent bioluminescent signal generation of **Pt‐Luc** in a mixed buffer where a PB (50 mM, pH 7.4) buffer containing 2 mM ascorbate was added to a Tris buffer (50 mM pH 7.4) containing luciferase (100 µg mL^−1^, Promega), 10 mM Mg^2+^, 0.1 mM Zn^2+^, and 2 mM ATP. Bioluminescent signals were measured at 37 °C for 2 h. c) HPLC chromatogram of **Pt‐Luc** in a mixed buffer where a PB (50 mM, pH 7.4) buffer containing 2 mM ascorbate was added to a Tris buffer (50 mM pH 7.4) containing 10 mM Mg^2+^, 0.1 mM Zn^2+^, and 2 mM ATP. d) A line graph showing the relationship between the increment of bioluminescence intensity and the percentage of Pt(IV) complex reduced. The reduction of Pt(IV) complexes was monitored in live cells using the probe **Pt‐Luc**. e) Experimental layout for real‐time monitoring of the reduction of **Pt‐Luc** in live cells. f) Time‐dependent bioluminescent signal generation of **Pt‐Luc** from A549‐Luc2 cells treated with diverse concentrations (0, 6.25, 12.5, 25, 50, and 100 µM) of **Pt‐Luc**. The increase in the bioluminescent signal slowed down as it approached the 180‐min mark; consequently, we concluded the measurement at this juncture. g) Bioluminescence imaging of A549‐Luc2 cells treated with diverse concentrations of **Pt‐Luc**. h) Bioluminescent signals from A549‐Luc2 cells probed with **Pt‐Luc**. Total photon flux was integrated over 3 h. Statistical analyses were performed using a two‐tailed Student's test, and error bars represent ± SD (n = 4).

A buffer‐based study was conducted to demonstrate the concept of employing bioluminescence to monitor the reduction process of Pt(IV) complexes. Aminoluciferin, but not the “caged” derivative, **Pt‐Luc**, exhibited bioluminescence signals in the buffer solution (Figure 1a). However, upon the addition of ascorbate, the caged substrates underwent cleavage during the reduction process, resulting in a gradual increase in bioluminescent intensity (Figure 1b). For rigorous quantification, the bioluminescence signals integrated at various time points were normalized to that at the 2‐h mark, where aminoluciferin was completely released from **Pt‐Luc**. Simultaneously, the reduction of the Pt(IV) complex was monitored using HPLC at designated intervals (Figure 1c). A linear relationship (R^2^ = 0.99425) was observed between the normalized bioluminescence intensity and amount of reduced Pt(IV) complexes determined by HPLC (Figure 1d), indicating that the light output effectively reflected the level of free aminoluciferin and the extent of Pt(IV) complex reduction. Remarkably, even at a minimal concentration of 10 nM for **Pt‐Luc**, a strong detectable bioluminescence signal was maintained, demonstrating a linear correlation (R^2^ = 0.98696) between the percentage of **Pt‐Luc** reduced and the normalized bioluminescence intensity (Figure S13), underscoring the high sensitivity of the **Pt‐Luc** reporter system.

We proceeded to assess whether the ability of **Pt‐Luc** to monitor the reduction in real time within an aqueous buffer could be extended to cellular environments, which offers the potential to visualize the reduction progress of Pt(IV) prodrugs in live cells. Initially, we examined the cellular accumulation of **Pt‐Luc**, and the results demonstrated favorable permeation of the “caged” substrates across the live cell membrane (Figure S14a). To ensure its applicability as a probe, we also conducted standard cytotoxicity assays to evaluate the biocompatibility of **Pt‐Luc**. The probe exhibited no cytotoxicity toward cells at concentrations up to 200 µM within a 3‐h timeframe (Figure S14b–d).

Subsequently, we investigated how **Pt‐Luc** responded to the reducing environment in live cells. A549‐Luc2 cells were exposed to varying concentrations of the probe, and continuous imaging was conducted for 180 min using a CCD camera (Figure 1e,f). The total photon flux was quantified by integrating the area under the time‐dependent bioluminescence curve. A dose‐dependent correlation was observed between the increasing concentrations of **Pt‐Luc** and corresponding increments in bioluminescence intensity (Figure 1g,h). The source of the observed bioluminescence in A549‐Luc2 cells treated with **Pt‐Luc** is intriguing, as it could arise from either the intracellular reduction or hydrolysis of **Pt‐Luc**.^[^ ^6^ ^]^ Using liquid chromatography‐mass spectrometry (LC‐MS), we confirmed the intracellular reduction of **Pt‐Luc** (Figure S15). Moreover, we did not detect the dihydroxido Pt(IV) compound [Pt(DACH)(OH)_2_(ox)], the hydrolyzed product from **Pt‐Luc**, thereby ascertaining that the bioluminescence signal indeed originates from the reduction process. Additionally, the minimum probe concentration required to observe specific signals above background labeling was determined to be as low as 0.5 µM (Figure S16). Collectively, these results, along with the high stability of the probe in the medium (Figure S10c,d), suggest that the aminoluciferin released from **Pt‐Luc** intracellularly contributes to the obtained bioluminescence signal. This indicates that the reduction process of Pt(IV) prodrugs in live cells can be sensitively detected utilizing this bioluminescence probe.

**Figure 2:**
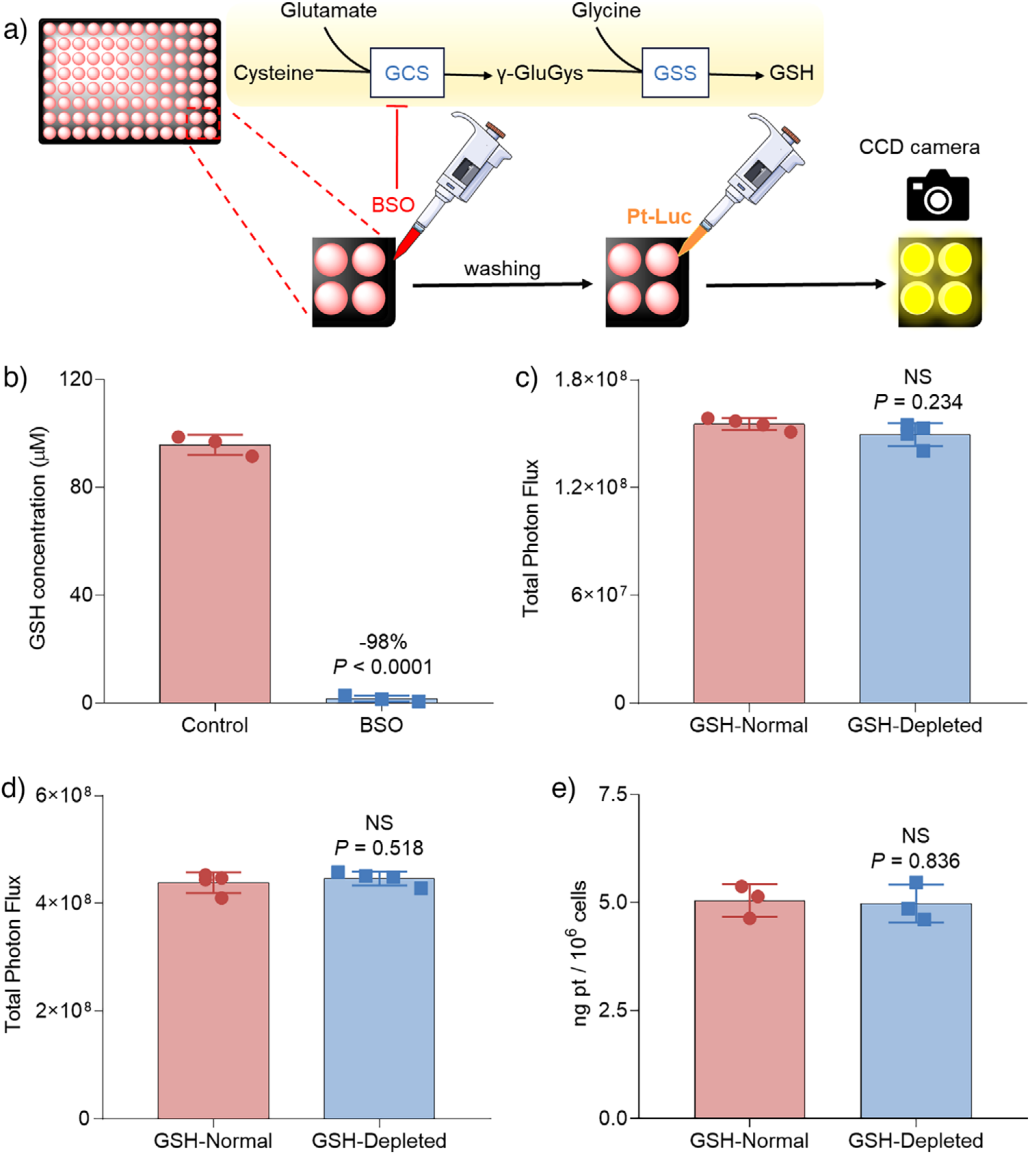
The application of **Pt‐Luc** to investigate the role of GSH in the reduction of Pt(IV) complexes in live cells. a) Experimental layout for measurements of BSO‐mediated bioluminescence signal production in A549‐Luc2 cells using the **Pt‐Luc** probe. A549‐Luc2 cells, initially treated with either BSO or PBS (control group) for 24 h, undergo continuous signal acquisition via a CCD camera upon treatment with **Pt‐Luc**. GSS, glutathione synthetase; γ‐GluGys, γ‐glutamylcysteine. b) The GSH content in A549‐Luc2 cells after preincubation of cells without or with BSO (250 μΜ for 24 h). Bioluminescent signals of **Pt‐Luc** c) and aminoluciferin d) from the GSH‐normal and GSH‐depleted A549‐Luc2 cells. Total photon flux was integrated over 3 h. e) Cellular accumulation of **Pt‐Luc** in GSH‐normal and GSH‐depleted A549‐Luc2 cells. Statistical analyses were performed using a two‐tailed Student's test, and error bars represent ± SD (**n** = 3).

Next, we employed **Pt‐Luc** to investigate the role of GSH in the reduction of Pt(IV) complexes within live cells. GSH was originally believed to be the primary reductant for Pt(IV) prodrugs due to its high concentration in cells (1–10 mM).^[^ ^31^ ^]^ We conducted an experiment where A549‐Luc2 cells were pretreated with buthionine‐sulfoximine (BSO), which effectively depleted GSH levels by inhibiting γ‐glutamylcysteine synthetase (GCS), a key enzyme in GSH synthesis (Figure 2a).^[^ ^36^, ^38^ ^]^ This facilitated the establishment of a cell pair model (GSH‐depleted vs. GSH‐normal) using A549‐Luc2 cells with and without BSO pre‐treatment (Figure 2b), allowing us to examine the impact of GSH on Pt(IV) prodrug reduction in live cells. When these cells were treated with **Pt‐Luc**, no statistical difference in bioluminescence intensities was observed between the GSH‐depleted and GSH‐normal A549‐Luc2 cells (Figure 2c). Control experiments using the parent aminoluciferin substrate confirmed that luciferase activity was not affected by GSH depletion (Figure 2d). Additionally, we assessed the accumulation levels of **Pt‐Luc** in GSH‐normal and GSH‐depleted A549‐Luc2 cells and found identical Pt contents (Figure 2e). The addition of exogenous GSH to the medium increased GSH levels in A549‐Luc2 cells but did not significantly influence the reduction of **Pt‐Luc** (Figure S17). Furthermore, a cell‐free study examining the reduction of **Pt‐Luc** by GSH showed that GSH was ineffective in reducing **Pt‐Luc** in PB buffer (50 mM, pH 7.4; Figure S18). These findings suggest that GSH may not play an essential role in the reduction of Pt(IV) complexes in live cells, aligning with observations from other studies using solution‐based assays, cell lysates, or fixed cells.^[^ ^35^, ^36^, ^38^, ^39^ ^]^

**Figure 3:**
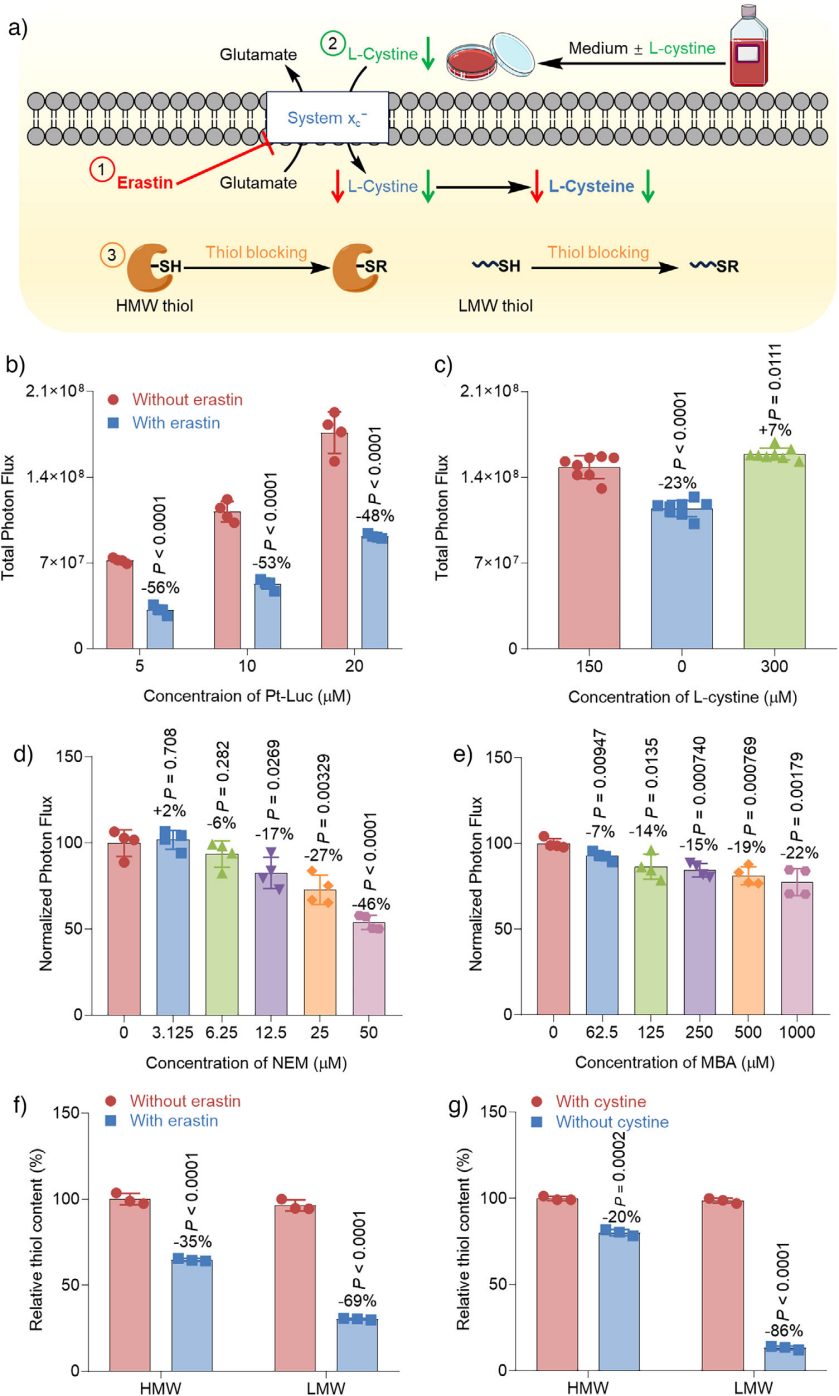
The application of **Pt‐Luc** to investigate the role of cysteine in the reduction of Pt(IV) complexes in live cells. The schematic demonstration includes three scenarios: a–1) erastin suppresses system $x_{c}$ ^−^ or a–2) L‐cystine depletion occurs in the medium, both of which thereby cause cysteine starvation; a–3) thiol blocking agents bind to the ‐SH group of the thiols, rendering them unavailable for further reactions. b) The total photon flux from A549‐Luc2 cells was measured after preincubating the cells with erastin (100 µM for 5 min), followed by washing and incubation for an additional 24 h. Subsequently, a fresh medium containing various concentrations of **Pt‐Luc** was added, and the total photon flux was integrated over 3 h. Statistical analyses were performed using a two‐tailed Student's test, and error bars represent ± SD (**n** = 4). c) The total photon flux from A549‐Luc2 cells was measured after preincubating the cells with L‐cystine‐normal, L‐cystine‐free, and L‐cystine‐excess medium for 24 h. Following the preincubation, the cells were washed, and a medium containing 20 µM of **Pt‐Luc** was added. Data are presented as mean ± SD, **n** = 8. The normalized photon flux was measured from A549‐Luc2 cells that were preincubated with varying concentrations of d) NEM and e) MBA for 0.5 h, followed by washing and addition of medium containing 20 µM of **Pt‐Luc**. f) The concentration of thiol levels in the high molecular weight (HMW) and low molecular weight (LMW) portions of the A549‐Luc2 cell extract were measured after pre‐treatment with erastin (100 µM for 5 min), followed by washing and incubation for an additional 24 h. g) The concentration of thiol levels in the HMW and LMW portions of the A549‐Luc2 cell extract were measured after treating the cells with L‐cystine normal and L‐cystine free medium, respectively, for 24 h.

Beyond its essential role in protein synthesis, cysteine plays a vital role in maintaining redox homeostasis and acts as a potent antioxidant in cells. It generates sulfur‐containing molecules such as GSH, taurine, lanthionine, and coenzyme A.^[^ ^40^, ^41^ ^]^ Given these roles, it would be reasonable to hypothesize that intracellular cysteine levels could affect the reduction of **Pt‐Luc** in live cells. System x_c_^−^ is a dedicated cystine transporter, facilitating the import of cystine in exchange for intracellular glutamate,^[^ ^40^, ^42^, ^43^ ^]^ and its activity can be inhibited by the small molecule erastin, leading to cysteine starvation within the cells (Figure 3a–1).^[^ ^44^ ^]^ We observed that both a short exposure (5 min) to a high concentration (100 µM) of erastin and a long pre‐treatment (6 h) with a low concentration (10 µM) of erastin effectively inhibited system x_c_^−^,^[^ ^45^, ^46^ ^]^ causing cysteine starvation (Figure S19a,b). Importantly, erastin did not significantly affect luciferase activity or cell viability under the experimental conditions (Figure S19c–f). Pretreating A549‐Luc2 cells with a high concentration of erastin significantly diminished the bioluminescent output from **Pt‐Luc**. Specifically, the bioluminescent signal was reduced by 56%, 53%, and 48% for concentrations of 5, 10, and 20 µM of **Pt‐Luc**, respectively (Figure 3b). Moreover, prolonged exposure to a lower concentration of erastin led to a 46%, 41%, and 34% decrease in bioluminescence intensity at 5, 10, and 20 µM of **Pt‐Luc**, respectively (Figure S20). Comparable levels of Pt were observed in erastin‐treated and untreated cells, eliminating the possibility that the reduced bioluminescence in erastin‐treated cells was due to the decreased uptake of **Pt‐Luc** (Figure S21). Furthermore, to clarify the role of cysteine in the reduction of Pt(IV) complexes in live cells, we examined the response of A549‐Luc2 cells to both cystine depletion and excessive supplementation (Figure 3a–2). Specifically, we assessed the responses of complex **Pt‐Luc** in A549‐Luc2 cells subjected to a medium with normal cystine levels, cystine depletion, and excess cystine supplementation (Figure S22a).^[^ ^47^ ^]^ Notably, cystine depletion resulted in a 23% decrease in the bioluminescence signal compared with the group with regular cystine levels, while cells supplemented with extra cystine showed a 7% increase in the bioluminescence signal (Figure 3c). Cystine had no significant effect on luciferase activity in any of the tested conditions (Figure S22b). We extended our investigation to MDA‐MB‐231‐Luc, a luciferase‐expressing breast cancer cell line, to further validate these findings. Similar to the observations in A549‐Luc2 cells, pre‐treatment with erastin or incubation in a cystine‐free medium resulted in diminished bioluminescence signals in MDA‐MB‐231‐Luc cells (Figure S23). These additional findings provide further support for the hypothesis that cysteine, as a component of sulfur‐containing molecules, plays an essential role in the intracellular reduction of Pt(IV) complexes.

The distinctive chemistry of the thiol group in cysteine imparts specialized properties such as nucleophilicity, high‐affinity metal binding, and the ability to form disulfide bonds.^[^ ^48^ ^]^ This leads us to question whether the concentration of thiols is the determining factor in the reduction process of Pt(IV) complexes. To investigate this, we exposed A549‐Luc2 cells to the thiol‐alkylating reagents *N*‐ethylmaleimide (NEM)^[^ ^49^, ^50^ ^]^ and maleimidobutyric acid (MBA)^[^ ^51^ ^]^ to block intracellular exposed thiols (Figure 3a‐3 and Figure S24). The reduction of complex **Pt‐Luc** in these pretreated cells decreased as the concentration of thiol‐blocking agents increased (Figure 3d,e). For example, pre‐treatment of cells with 12.5 and 50 µM of NEM resulted in a decrease of 17% and 46% in the bioluminescence signal, respectively. These results provide evidence for the substantial contribution of intracellular thiols in the reduction of Pt(IV) complexes.

Thiols in biological systems can be classified into two primary groups: protein thiols, referred to as HMW thiols, and non‐protein thiols, known as LMW thiols.^[^ ^48^, ^52^ ^]^ When A549‐Luc2 cells were treated with erastin or exposed to a cystine‐free medium, the levels of both HMW and LMW thiols decreased (Figure 3f,g). This raises the question of which component, HMW or LMW thiols, contributes more significantly to the reduction of the Pt(IV) complex.

**Figure 4:**
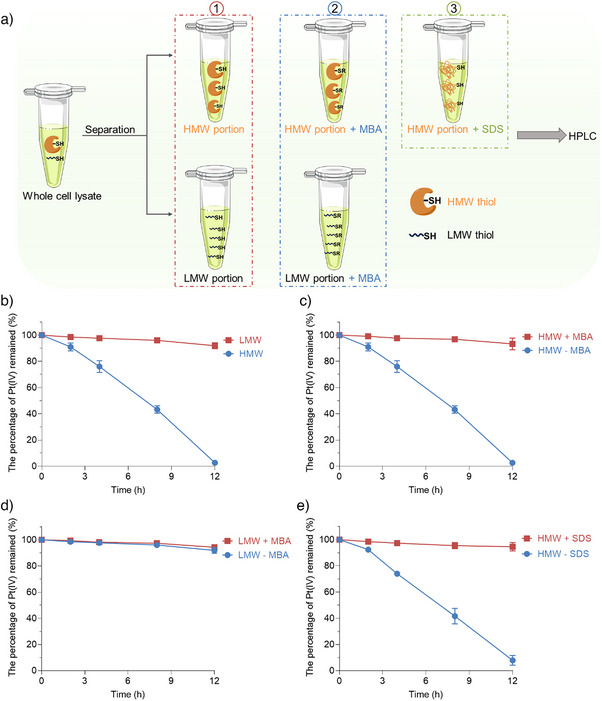
a–1) The reduction of **Pt‐Luc** in high molecular weight (HMW) and low molecular weight (LMW) portions, separated from the A549‐Luc2 cell extracts, was measured by HPLC. a–2) The same measurement was performed on HMW and LMW portions where thiol groups, blocked by MBA, became unavailable for further reactions. a–3) The reduction of **Pt‐Luc** was also measured in the HMW portion, where proteins were denatured by sodium dodecyl sulfate (SDS). b) The percentage of remaining Pt(IV) complex **Pt‐Luc** in the HMW and LMW portions. The initial concentrations of thiols in HMW and LMW fractions were adjusted to the same level. c) The percentage of remaining Pt(IV) complex **Pt‐Luc** in the HMW portion with and without MBA. d) The percentage of remaining Pt(IV) complex **Pt‐Luc** in the LMW portion with and without MBA. e) The percentage of remaining Pt(IV) complex **Pt‐Luc** in the HMW portion with and without SDS.

To determine the contribution of different thiol fractions to the reduction of Pt(IV) complexes, we fractionated A549‐Luc2 cell extracts to yield HMW and LMW thiols (Figure S25). Subsequently, we monitored the reduction of **Pt‐Luc** in each fraction by HPLC (Figure 4a–1). The HMW protein fraction demonstrated significantly greater efficiency in activating **Pt‐Luc** than the LMW fraction (Figure 4b), consistent with previous studies.^[^ ^35^, ^36^ ^]^ Blocking HMW thiols with MBA dramatically inhibited the reduction of the probe (Figures 4a–2, c and Figure S26a), whereas pre‐treatment of LMW thiols with MBA had minimal effects on its reduction, despite the inherently low reduction capacity of LMW thiols (Figure 4d and Figure S26b). Furthermore, the HMW fractions from erastin‐pretreated cells or those exposed to the cystine‐free medium showed a diminished capacity to reduce **Pt‐Luc** compared with the HMW fractions from the erastin‐free and cystine‐normal groups, respectively (Figure S27). These findings collectively suggest that protein cysteines, present in the HMW portion, play a crucial role in the reduction process. To confirm the contribution of protein cysteine residues to the reduction process observed in the HMW fraction, we subjected the HMW portion to sodium dodecyl sulfate (SDS) for protein denaturation (Figure 4a–3).^[^ ^53^, ^54^ ^]^ Remarkably, the presence of the protein denaturant resulted in a significant decrease in the reduction of **Pt‐Luc** (Figure 4e), while the thiol content did not decrease (Figure S28). These results indicate the essential role of protein cysteines in the reduction of Pt(IV) prodrugs.^[^ ^55^, ^56^ ^]^

**Figure 5:**
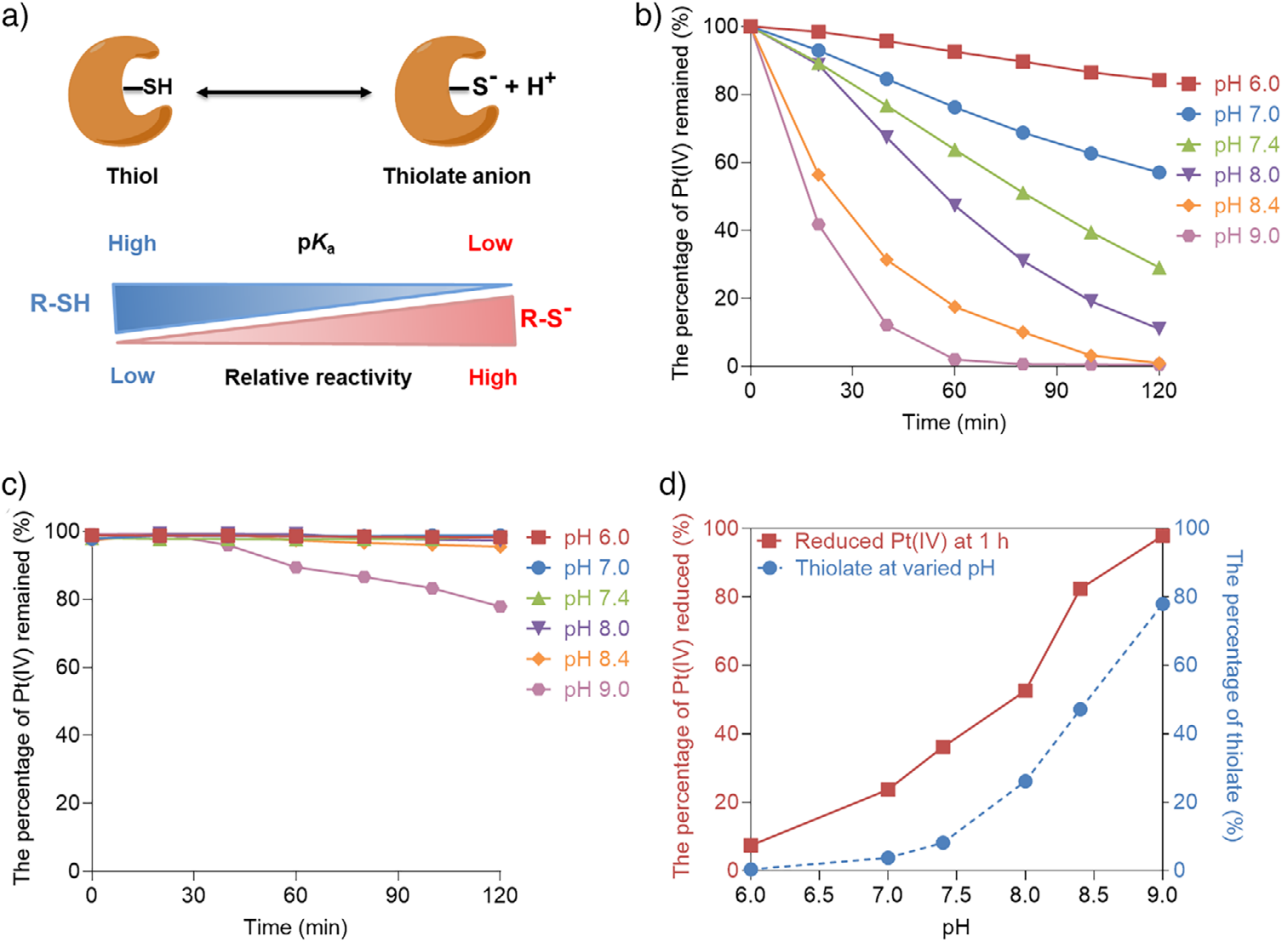
a) The relationship between thiol p*K*_a_ and the reactivity of thiolate in nucleophilic attack. The acid dissociation constant (logarithmic form, p*K*_a_) of a thiol group determines the ratio of thiol to thiolate at a given pH. This value varies for each cysteinyl thiol and is influenced by the local protein environment. The thiol with a lower p*K*_a_ is, therefore, more reactive than thiols with higher p*K*_a_ values. The reduction and stability of **Pt‐Luc** in PB buffer were measured by HPLC. b) The percentage of remaining Pt(IV) complex **Pt‐Luc** during the incubation in a PB buffer (50 mM, with varying pH levels) at 37 °C containing 2 mM cysteine. c) The percentage of remaining Pt(IV) complex **Pt‐Luc** during the incubation in a PB buffer (50 mM, with varying pH levels) at 37 °C. d) The percentage of Pt(IV) complex **Pt‐Luc** reduced at 1 h in a PB buffer (50 mM, with varying pH levels) at 37 °C containing 2 mM cysteine was plotted against different pH levels (solid line); the percentage of thiolate (S^−^) at different pH values was calculated using the Henderson–Hasselbalch equation (pH = p*K*_a_ + log([A^−^]/[HA]) (dotted line).

The thiol group in cysteines plays a crucial role in various nucleophilic reactions within biochemical systems.^[^ ^57^, ^58^ ^]^ The reactivity of the sulfhydryl group, which is contingent upon its p*K*_a_ value, increases with a decrease in p*K*_a_ due to the deprotonated thiolate form (RS^−^) exhibiting enhanced nucleophilicity and heightened reactivity with oxidants compared with the protonated form (R‐SH) (Figure 5a).^[^ ^59^ ^]^ In a thiol‐disulfide exchange reaction, for instance, the thiolate anion RS^−^ reacts approximately 10^10^ times faster than the corresponding thiol.^[^ ^60^ ^]^ In free cysteine, the thiol group has a p*K*_a_ of 8.45, meaning that only a small fraction of cysteines undergo deprotonation at physiological pH, rendering them relatively inert for redox reactions in the physiological environment.^[^ ^57^, ^58^ ^]^ However, the p*K*_a_ values of cysteine thiols in proteins are dramatically influenced by their local environment. Although the average p*K*_a_ values of cysteines buried in the protein core are approximately 9.5, cysteines located in active sites can have significantly lower p*K*_a_ values, as low as 3.4. This can be attributed to the stabilizing effects of the protein environment, including positively charged amino acids, helix dipoles, and coordination with metal ions like Zn^2+^ and Fe^2+/3+^, on the negatively charged thiolate anion.^[^ ^48^, ^57^, ^58^, ^59^, ^60^ ^]^ As the thiolate form is a more potent nucleophilic agent than its protonated form, reactive cysteines in proteins predominantly exist as anionic species. These reactive cysteine residues exhibit highly versatile redox chemistry, with their sulfur atom capable of transitioning between oxidation states ranging from +6 to −2. They possess the ability to engage in electron transfer^[^ ^59^ ^]^ and can thus reduce Pt(IV) complexes. Hence, it is reasonable to hypothesize that active cysteines in proteins serve as the dominant reducing agents responsible for Pt(IV) reduction in live cells.

To investigate our hypothesis regarding the higher nucleophilicity and reactivity of active cysteine (RS^−^) in contributing to the reduction of **Pt‐Luc**, we conducted experiments using PB buffer supplemented with cysteine at varying pH levels. We observed a notable acceleration in the reduction of **Pt‐Luc** with increasing pH (Figure 5b), consistent with the deprotonated species being more effective reductants.^[^ ^21^, ^61^, ^62^ ^]^ Despite these pH changes, most of the **Pt‐Luc** maintained its integrity (Figure 5c). This relationship is further supported by the observation that the calculated amount of thiolate directly correlates with the extent of **Pt‐Luc** reduction (Figure 5d), confirming the enhanced reactivity of thiolate. Collectively, these findings indicate that the reactive cysteines present in proteins act as the dominant reducing agents for Pt(IV) prodrugs in live cells.

**Figure 6:**
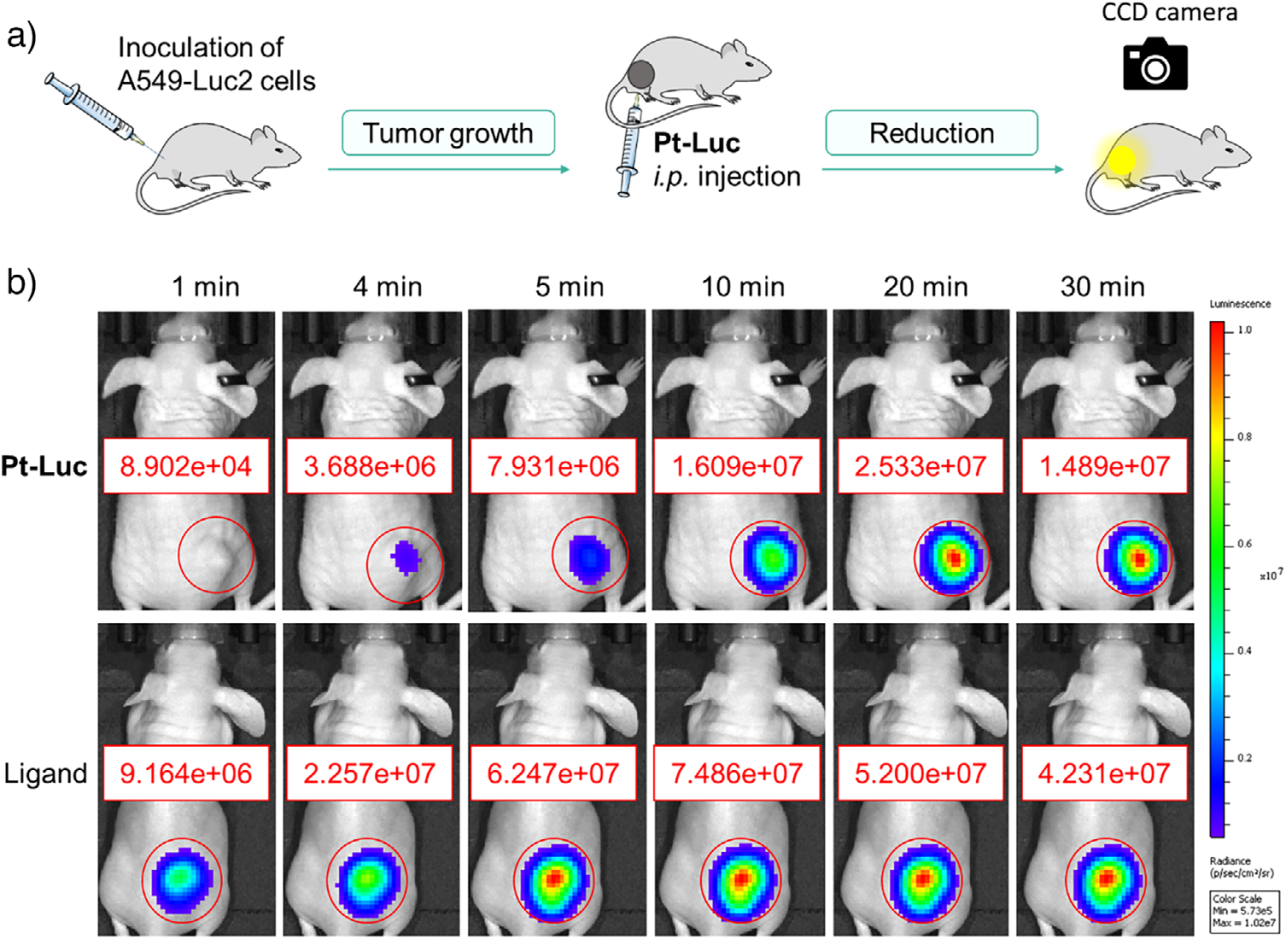
a) Experimental layout for measurements of reduction‐mediated light production in mice using the **Pt‐Luc** probe. b) Representative images of Balb/c nude mice bearing A549‐Luc2 tumor xenografts were administered with either **Pt‐Luc** (50 µM, 200 µL per 20 g mouse body weight; upper) or aminoluciferin (50 µM, 200 µL per 20 g mouse body weight; lower) at different time points. The bioluminescent area of the xenograft tumor, indicated by a red circle, was defined as the region of interest (ROI); the red number within the rectangle represents the total signal detected in the ROI, quantified using Living Image software 3D.

Previous research on the reduction of Pt(IV) prodrugs in vivo has often been constrained by the lack of real‐time monitoring capabilities, necessitating multiple sampling time points.^[^ ^39^ ^]^ Bioluminescence emerges as a noninvasive, real‐time monitoring tool, positioning it among the selected methods capable of tracking Pt(IV) reduction in animal models. Additionally, bioluminescence imaging provides high‐sensitivity detection and functions independently without the need for external light sources. These combined advantages render it an ideal approach for investigating Pt(IV) reduction in vivo. Consequently, the utility of the probe **Pt‐Luc** was further extended to in vivo monitoring. We first subcutaneously injected A549‐Luc2 cells into Balb/c nude mice and subsequently performed imaging using **Pt‐Luc** and aminoluciferin (Figure 6a). In the mouse treated with **Pt‐Luc**, a weak emission signal gradually emerged, contrasting with the immediate intense signal from the aminoluciferin‐treated mouse (Figure 6b), indicating a diminished bioluminescence activity due to the delayed release of free aminoluciferin from the “caged” **Pt‐Luc**, showcasing **Pt‐Luc**’s ability to monitor the reduction of Pt(IV) complexes in vivo. Additionally, our assessment of **Pt‐Luc**’s stability in blood indicated that the complex remained intact during transport from the peritoneal cavity to the systemic circulation (Figure S29) and largely maintained its integrity within 30 min (Figure S30), suggesting that although some activation may occur prior to reaching the tumor site, the primary activation predominantly takes place at the tumor site, significantly contributing to the observed bioluminescence. The reduction profiles at tumor sites in vivo (Figure S31a) differ from those observed in live cells (Figure S31b), likely due to the unique characteristics of the tumor microenvironment and the complexities inherent in living systems. This disparity highlights the differences between in vitro and in vivo studies, underscoring the essential need for assessing the activation of **Pt‐Luc** in live animals.

**Figure 7:**
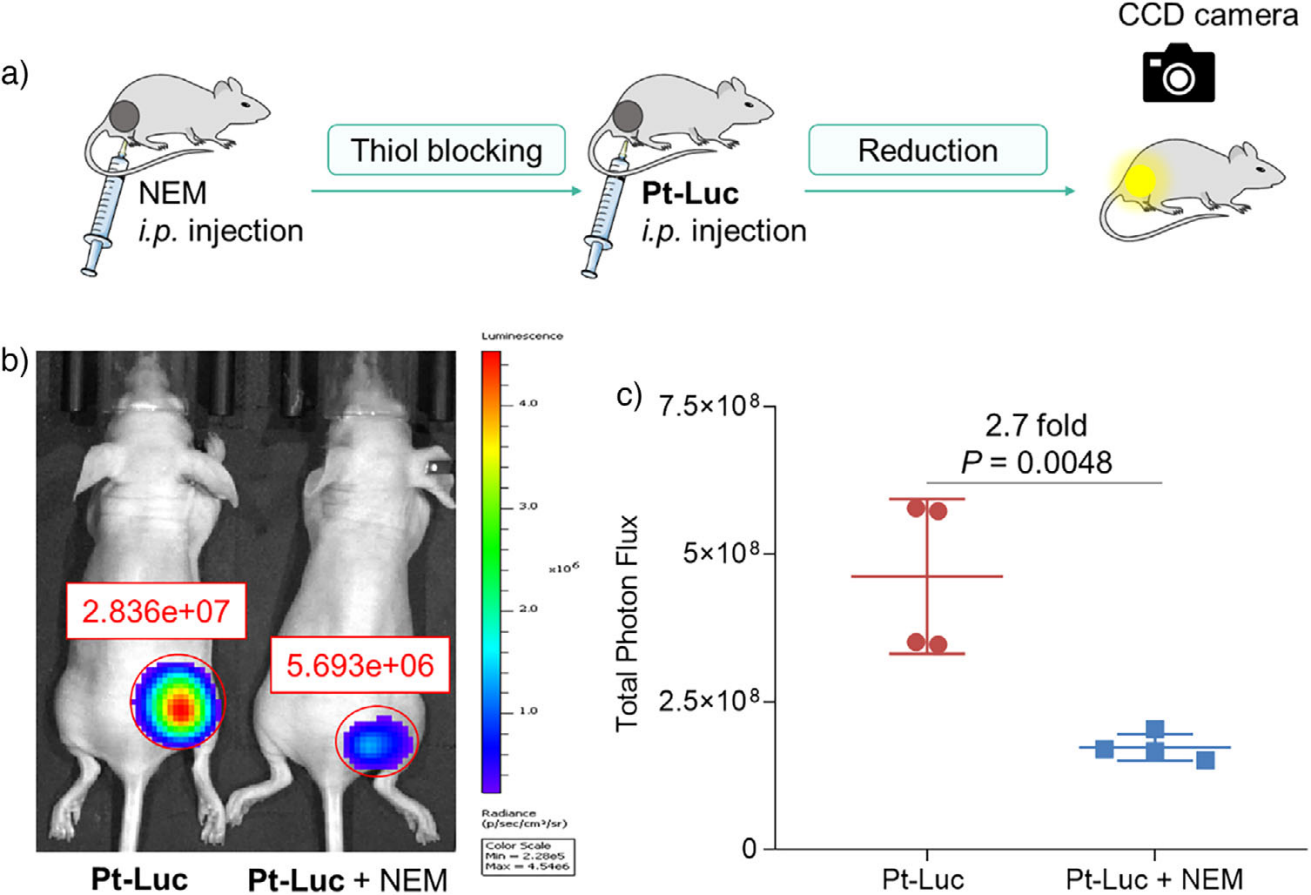
a) Experimental layout for investigating the influence of thiol levels on the reduction of **Pt‐Luc** in mice. These mice were initially administered with either vehicle or NEM, followed by **Pt‐Luc** injection (50 µM, 200 µL per 20 g mouse body weight) 30 min later. b) Representative images of Balb/c nude mice bearing A549‐Luc2 tumor xenografts were administered with either **Pt‐Luc** only (left) or **Pt‐Luc** with NEM (right) at 16 min. All presented experiments were repeated thrice. c) Total photon flux, integrated over 30 min, from cancer‐bearing mice injected with **Pt‐Luc** ± NEM. Data are presented as mean ± SD.

After establishing the system's feasibility, we investigated the role of active protein thiols in activating Pt(IV) prodrugs in vivo using the **Pt‐Luc** probe, a role substantiated through in vitro studies mentioned above. The mice were administered an intraperitoneal injection of the thiol‐blocking agent NEM to inhibit exposed thiols in the tumor (Figure 7a and Figure S32). Following a 30‐min interval, the mice were intraperitoneally injected with **Pt‐Luc** or aminoluciferin and immediately imaged. Strikingly, despite receiving the same dosage, the bioluminescent signal in the tumors of the NEM‐treated group was remarkably diminished compared with that in the untreated group (Figure 7b). Importantly, the activity of luciferase remained unaffected by NEM treatment (Figure S33). The total luminescent count for the NEM‐treated group was only 1.7 ± 0.2 × 10^8^, significantly lower than that (4.6 ± 1.3 × 10^8^) for the untreated group (Figure 7c). These results demonstrate a significant decrease of 63% in the bioluminescence signal upon NEM treatment, underscoring the importance of protein thiols in activating Pt(IV) prodrugs in live mice.

## Conclusion

In conclusion, we developed **Pt‐Luc**, a bioluminescence probe equipped with a luciferase substrate, for real‐time tracking of Pt(IV) prodrug reduction in live cells. Upon reduction, the probe liberated aminoluciferin, which was subsequently oxidized by luciferase, resulting in light emission. This emitted light, captured and quantified in real time, correlated linearly with the reduction percentage of **Pt‐Luc**, thereby enabling the quantification of the reduction through bioluminescence monitoring. Using **Pt‐Luc**, we investigated the reduction of Pt(IV) prodrugs in live cells and made several important discoveries. The reduction of Pt(IV) prodrugs remained consistent, regardless of GSH depletion. However, a significant attenuation of the reduction was observed when protein thiol levels decreased. Reactive cysteines in proteins were identified as the primary agents responsible for this reduction. This was further substantiated in a murine lung cancer model, where in vivo imaging with **Pt‐Luc** showed a significant decrease in bioluminescence in thiol‐blocking mice. Overall, this study reports a bioluminescent probe **Pt‐Luc** that enables real‐time and noninvasive monitoring of Pt(IV) reduction in live cells and animal models. These findings not only enhance our understanding of the activation mechanisms of Pt(IV) prodrugs in live cells and in vivo but also have potential applications for future research and therapeutic developments.

## Conflict of Interests

The authors declare no conflict of interest.

## Supporting information



Supporting Information

## Data Availability

The data that support the findings of this study are available from the corresponding author upon reasonable request.
