# Supplementary material for: Reactive Cysteines in Proteins are the Dominant Reductants for Platinum(IV) Prodrug Activation in Live Cells
Source: Angew Chem Int Ed Engl. 2025 Apr 26;64(25):e202416396. doi: 10.1002/anie.202416396 (PMC12171337; doi:10.1002/anie.202416396)
Supplement: Supplementary file 1 — Supporting Information [file ANIE-64-e202416396-s001.pdf]

Supporting Information
©Wiley-VCH 2021
69451 Weinheim, Germany

Reactive Cysteines in Proteins Are the Dominant Reductants for Platinum(IV) Prodrug Activation in Live Cells

Shu Chen, Wang Peng, Houzong Yao, Zhiqin Deng, Zhao Yue, Gongyuan Liu, Jiaqian Xu, Naixin Lin, Weikang Xu, Jianbo Yue, and Guangyu Zhu*

Abstract: The intracellular reduction of Pt(IV) prodrugs is crucial for their anticancer efficacy. However, the major components responsible for the reduction of Pt(IV) complexes within live cells remain elusive. Herein, we developed an aminoluciferin-functionalized Pt(IV) complex, **Pt-Luc**, that can be used as a bioluminescent reporter for real-time monitoring of Pt(IV) reduction in live cells by capturing immediate bioluminescent signals from the released aminoluciferin. Utilizing this powerful reporter, we found that the reduction of Pt(IV) prodrugs in live cells significantly slows down when cysteine levels are reduced, while the levels of glutathione do not impact the reduction rate. Further investigation reveals that reactive cysteines in proteins, rather than small-molecule thiols, play a primary role in reducing the Pt(IV) complex. In vivo studies reveal a substantial 63% decrease in bioluminescence from **Pt-Luc** in thiol-blocking tumors in mice, reinforcing the pivotal role of reactive cysteines in Pt(IV) reduction. This study provides valuable insights into the activation mechanisms of Pt(IV) prodrugs in live cells and in vivo, enhancing our understanding of prodrug activation beyond buffer systems or fixed cells.

DOI: 10.1002/anie.2021XXXXX

Table of Contents

Experimental procedures
Scheme S1. Synthetic route for bioluminescent probe **Pt-Luc**
Figures S1-S4. ESI-HRMS, ^{1}H NMR, ^{13}C NMR, and ^{195}Pt NMR spectra of complex **1c**
Figures S5-S8. ESI-HRMS, ^{1}H NMR, ^{13}C NMR, and ^{195}Pt NMR spectra of complex **Pt-Luc**
Figure S9. HPLC chromatogram for the purity test of **Pt-Luc**
Figure S10. The stability and reduction tests of the bioluminescent Pt(IV) probe
Figures S11-S12. The ESI-Mass spectrum of reduction products with a retention time of 10.8 min and 6.8 min, respectively
Figure S13. The detection limit of Pt(IV) complex **Pt-Luc** in buffer solution
Figure S14. The cellular accumulation and the MTT test of complex **Pt-Luc** and oxaliplatin
Figure S15. The reduction products of **Pt-Luc** in A549-Luc2 cells
Figure S16. The detection limit of Pt(IV) complex **Pt-Luc** in live cells
Figure S17. The application of **Pt-Luc** to investigate the role of exogenous GSH addition in the reduction of Pt(IV) complexes in live cells.
Figure S18. The percentage of remaining Pt(IV) complex **Pt-Luc** in a PB buffer containing 2 mM GSH or L-cysteine
Figure S19. The cysteine content, luciferase activity, and cell viability of A549-Luc2 cells after being treated with erastin
Figure S20. Total photon flux from A549-Luc2 cells after preincubation of cells with or without erastin
Figure S21. The cellular accumulation of **Pt-Luc** in A549-Luc2 cells after preincubation with or without erastin
Figure S22. The cysteine content and luciferase activity of A549-Luc2 cells after being treated with different levels of L-cystine
Figure S23. The application of **Pt-Luc** to investigate the role cysteine plays in the reduction of Pt(IV) complexes in MDA-MB-231 Luc cells
Figure S24. The cell viability and thiol levels of A549-Luc2 cells after being treated with NEM or MBA
Figure S25. The protein and GSH concentrations in the high molecular weight (HMW) and the low molecular weight (LMW) portions
Figure S26. The thiol levels in HMW and LMW portions with or without treatment with MBA
Figure S27. The percentage of remaining Pt(IV) complex **Pt-Luc** in the HMW portion of A549-Luc2 cell extracts
Figure S28. The thiol levels in the HMW portion that were pretreated with or without SDS
Figure S29. Stability assessment of **Pt-Luc** in blood following intraperitoneal (i.p.) versus intravenous (i.v.) injection in BALB/c nude mice
Figure S30. The percentage of remaining Pt(IV) complex **Pt-Luc** in blood
Figure S31. The reduction profiles of **Pt-Luc** in vivo and in live cells
Figure S32. The thiol concentrations in tumors with or without treatment of NEM
Figure S33. The in vivo study for investigating the influence of thiol levels on the activity of luciferase in mice
Reference
Author Contributions

Experimental Procedures

Materials and instruments

Unless stated otherwise, all chemicals and solvents were procured from authentic commercial suppliers and used without further purification. All the reactions were carried out under normal atmospheric pressure, under the exclusion of light by tin foil. Ham's F-12K (Kaighn's) medium, Dulbecco's modified eagle medium (DMEM), Roswell Park Memorial Institute (RPMI) 1640 medium, trypsin, phosphate-buffered saline (PBS, pH 7.4), penicillin and streptomycin, sodium pyruvate, and fetal bovine serum (FBS) were obtained from ThermoFisher.

A549-Luc2 (ATCC CCL-185-LUC2) cell line was obtained from the American Type Culture Collection (ATCC), Manassas, VA, USA. MDA-MB-231-Luc was a generous gift from Prof. Jianbo Yue (Department of Biomedical Sciences, City University of Hong Kong).

The newly synthesized Pt(IV) complexes were characterized by ^{1}H NMR, ^{13}C NMR, ^{195}Pt NMR spectroscopy, and electrospray ionization high-resolution mass spectrometry (ESI-HRMS). All NMR spectra were obtained by a Bruker AVANCE III 400 MHz spectrometer or a Bruker Ascend AVANCE III 600 MHz spectrometer at room temperature. Chemical shifts in the ^{1}H and ^{13}C NMR spectra were referenced with the individual solvent residual peak of deuterated dimethyl sulfoxide (DMSO-d_6). Chemical shifts of ^{195}Pt NMR spectra were reported with respect to the chemical shift of K_2PtCl_4 in D_2O (δ = −1628 ppm vs Na_2PtCl_6). The ESI-HRMS was carried out by an HPLC-High Resolution Mass Spectrometer (Sciex X500R Q-TOF). The reverse-phase high-performance liquid chromatography (RP-HPLC) experiments were performed on a Shimadzu Prominence HPLC System equipped with a Phenomenex C18 column (5 µm, 110 Å, 250 × 4.6 mm^2) to monitor the purity, stability, and reduction of the synthetic complexes. For the purification of complexes, a Waters semi-preparative column (XBridge BEH C18 OBD Prep Column, 130 Å, 5 µm, 250 mm ×10 mm) was utilized. The platinum content was measured by a PerkinElmer Optima 8000 inductively coupled plasma optical emission spectrometer (ICP-OES) and a PerkinElmer 2000 inductively coupled plasma mass spectrometry (ICP-MS). Both of them were set to yield a maximum sensitivity for platinum. The Molecular Devices SpectraMax ID5 Microplate Reader was used to record the absorption or bioluminescence. Bioluminescence imaging was conducted using a Lumina In Vivo Imaging System (IVIS) Spectrum instrument (PerkinElmer).

Synthesis and characterizations

Synthesis of 1c. 4-nitrophenyl chloroformate (90 mg, 0.446 mmol) was dissolved into a solution of ethyl acetate (10 mL). To this solution, **1a** (50 mg, 0.285 mmol) was added, and stirring was maintained at room temperature overnight.[1] The solvent was then evaporated by reduced pressure, yielding the crude **1b**, which was used in the subsequent step without further purification. *t*-[Pt(DACH)(ox)(OH)$_2$] (50 mg, 0.116 mmol) and **1b** (36 mg, 0.104 mmol) were combined in 5 mL of dry DMSO. The resulting reaction mixture was stirred at room temperature for 48 h. After centrifugation to remove the unreacted *t*-[Pt(DACH)(ox)(OH)$_2$], the supernatant was added dropwise to a large volume of a mixture of acetone/DCM/Et_2O. A light-yellow precipitate of **1c** was collected by centrifugation and washed with DCM/Et_2O twice. The pure **1c** was obtained through lyophilization following RP-HPLC purification. ^{1}H NMR (600 MHz, DMSO-d_6) δ 9.51 (s, 1H), 8.71 (s, 1H), 8.45 (s, 1H), 8.23 (s, 1H), 8.07 (d, J = 9.1 Hz, 1H), 7.90 (s, 1H), 7.62 (d, J = 8.7 Hz, 1H), 7.21 (s, 1H), 2.70 – 2.62 (m, 2H), 2.15 – 2.07 (m, 2H), 1.57 – 1.41 (m, 4H), 1.14 (t, J = 9.5 Hz, 2H); ^{13}C NMR (151 MHz, DMSO-d_6) δ 163.9, 161.7, 146.3, 141.8, 136.9, 133.1, 124.5, 120.1, 113.7, 108.9, 61.3, 60.2, 30.9, 30.7, 23.7; ^{195}Pt NMR (129 MHz, DMSO-d_6) δ 1422.9 ppm. ESI-HRMS (positive ion mode) m/z: [M + H]$^+$ calculated for $C_{17}H_{20}N_5O_7PtS$ 633.0731, found 633.0625.

***Synthesis of t-[Pt(DACH)(ox)(OH)aminoluciferin]* (Pt-Luc).** A solution of complex **1c** (40 mg, 0.063 mmol) in a mixture of MeOH and H_2O (10 mL, v/v = 1:1) was combined with D-cysteine (10.8 mg, 0.089 mmol) and K_2CO_3 (12 mg, 0.089 mmol).[2] The reaction mixture was stirred under an argon atmosphere, and the progression of the reaction was monitored by RP-HPLC. Upon completion, the methanol was evaporated under reduced pressure, and the remaining aqueous solution was acidified with diluted HCl, yielding the crude product **Pt-Luc** as a yellow solid. The pure **Pt-Luc** was obtained by lyophilization following RP-HPLC purification. Yield: 16.7 mg (36%). Purity = 97% as determined by RP-HPLC at 340 nm. ^{1}H NMR (600 MHz, DMSO-d_6) δ 9.36 (s, 1H), 8.81 (s, 1H), 8.28 (d, *J* = 48.7 Hz, 2H), 8.08 – 7.82 (m, 2H), 7.50 (d, *J* = 8.4 Hz, 1H), 7.24 (s, 1H), 5.28 (s, 1H), 3.68 (dd, *J* = 9.0, 5.3 Hz, 2H), 2.74 – 2.62 (m, 2H), 2.12 (t, *J* = 12.4 Hz, 2H), 1.61 – 1.34 (m, 4H), 1.12 (d, *J* = 28.0 Hz, 2H); ^{13}C NMR (151 MHz, DMSO-d_6) δ 171.2, 163.9, 163.0, 161.9, 157.9, 147.3, 140.4, 136.4, 123.8, 118.8, 109.3, 79.7, 61.3, 60.2, 35.1, 30.9, 30.7, 23.7; ^{195}Pt NMR (129 MHz, DMSO-d_6) δ 1422.1. ESI-HRMS (positive ion mode) m/z: [M + H]$^+$ calculated for $C_{20}H_{24}N_5O_9PtS_2$ 737.0663, found 737.0686.

Measurement of stability and reduction of the bioluminescent probe

Stability in PB buffer. Pt(IV) complex **Pt-Luc** (5 μM, 0.4% DMF) was incubated in a PB buffer (50 mM, at varying pH levels). The incubation was carried out at 37 °C with protection from light. At scheduled time points, 100 μL samples were injected into the RP-HPLC system to track the degradation of the starting material. The percentage of the remaining Pt(IV) complex was normalized to that of time zero.

Stability in a cell culture medium. A solution of 50 μM of complex **Pt-Luc** was prepared using an F-12K medium, with 0.4% DMF as the supporting solvent. The solutions were incubated at 37 °C in a water bath. Prior to HPLC injection, the aliquot solution was mixed with four volumes of methanol and centrifuged at 14,000 rpm for 5 minutes to remove proteins. 100 μL of supernatant was then injected into the HPLC system to monitor the decay of the corresponding starting material.

Reduction test in PB buffer using ascorbate as the reducing agent. Pt(IV) complex **Pt-Luc** (5 μM, 0.4% DMF) was incubated in a PB buffer (50 mM, pH 7.4) containing 2 mM ascorbate. The solutions were incubated at 37 °C under light protection. At scheduled time points, 100 μL samples were injected into the RP-HPLC system. The percentage of the remaining Pt(IV) complex was normalized to that of time zero. The identities of the reduction products were determined by comparing their retention time with that of authentic standards and by utilizing electrospray ionization−mass spectrometry (ESI−MS) analysis.

Reduction test in PB buffer using glutathione(GSH) as the reducing agent. Pt(IV) complex **Pt-Luc** (5 μM, 0.4% DMF) was incubated in a PB buffer (50 mM, pH 7.4) containing 2 mM GSH. The solutions were incubated at 37 °C under light protection. At scheduled time points, 100 μL samples were injected into the RP-HPLC system. The percentage of the remaining Pt(IV) complex was normalized to that of time zero.

Reduction test in PB buffer using L-cysteine as the reducing agent. Pt(IV) complex **Pt-Luc** (5 μM, 0.4% DMF) was incubated in a PB buffer (50 mM, at varying pH levels) containing 2 mM L-cysteine. The solutions were incubated at 37 °C under light protection. At scheduled time points, 100 μL samples were injected into the RP-HPLC system. The percentage of the remaining Pt(IV) complex was normalized to that of time zero.

The HPLC wavelength for complex **Pt-Luc** was set as 340 and 350 nm. Mobile phases consisting of solvent A (H_2O with 0.1% formic acid) and solvent B (acetonitrile with 0.1% formic acid) were employed. The samples were eluted using the following program: 10% B (0 min) → 40% B (3min) → 52% B (15 min) → 10% B (16 min) → 10% B (20 min). The flow rate was maintained at 1.0 mL/min.

***In vitro* bioluminescent assays**

Incubation of **Pt-Luc** (5 μM, 0.4% DMF) was performed in 50 mM PB buffer at pH 7.4, supplemented with 2 mM ascorbate. 50 μL of a solution containing 20 mM Mg^{2+} ($MgCl_2$), 0.2 mM Zn^{2+} ($ZnCl_2$), and 4 mM ATP in 50 mM Tris buffer at pH 7.4 was transferred to a well of a white, opaque 96-well plate (Corning). Subsequently, 50 μL of a luciferase solution (200 μg/ml, Promega) in 50 mM Tris buffer was added and thoroughly mixed. [2-3] Then, 100 μL of the solution of **Pt-Luc** was injected into the plate using an injector. Bioluminescent signals were measured at 37°C for 2 h using a Molecular Devices SpectraMax ID5 Microplate Reader. Simultaneously, a 100 μL solution of 5 μM **Pt-Luc** in PB buffer (50 mM, pH 7.4) with 2 mM ascorbate was combined with a 100 μL solution containing 10 mM Mg^{2+} ($MgCl_2$), 0.1 mM Zn^{2+} ($ZnCl_2$) and 2 mM ATP in 50 mM Tris buffer at pH 7.4. The mixture was thoroughly mixed, incubated at 37 ℃, and subsequently injected into HPLC at predetermined time points to monitor the degradation of complex **Pt-Luc**.

Measurement of cellular accumulation

A549-Luc2 (4 × 10^5 per well) cells were seeded in 6-well plates and cultured at 37 °C for 48 h. Subsequently, the cells were exposed to oxaliplatin, *t*-[Pt(DACH)(ox)(OH)($OOCCH_3$)] (Ac-OxaPt(IV)-OH), and **Pt-Luc** at predetermined time intervals. After carefully removing the media, the cells were washed thrice with ice-cold PBS and harvested using trypsinization (0.5 mL of trypsin per well). The obtained cell suspensions were centrifuged at 1000 g for 5 minutes at 4 °C and subsequently washed twice with 4 mL of cold PBS. Cell counting was performed using a hemocytometer. The platinum concentration within the cells was determined by an ICP-MS after digestion with nitric acid at 80 °C for 24 hours and subsequent dilution with milli-Q water to a final volume of 1 mL. The data represent three independent measurements and are expressed as ng Pt per 10^6 cells.

Measurement of cell viability by MTT

Check whether Pt-Luc is toxic to A549-Luc2 cells within a short time. A549-Luc2 cells (2 x 10^4 per well) were seeded into 96-well plates and incubated at 37 ℃ for 48 h. The cells were treated with varying concentrations of **Pt-Luc** (1% DMF) in an FBS-free medium

and incubated for different time intervals. Alternatively, the cells were treated with 200 μM of oxaliplatin, *t*-[Pt(DACH)(ox)(OH)($OOCCH_3$)] (Ac-OxaPt(IV)-OH), and **Pt-Luc** (1% DMF), respectively, in the FBS-free medium for 3 h. After that, the cells were washed thrice with phosphate-buffered saline (PBS). Subsequently, 200 μL of a solution containing 3-(4,5-dimethylthiazol-2-yl)-2,5-diphenyltetrazolium bromide (MTT) at a concentration of 1.0 mg/mL was added, followed by incubation for an additional 2 hours at 37 °C. The MTT solution was then carefully removed, and 200 μL of dimethyl sulfoxide (DMSO) was added to each well to dissolve the formed crystals. The absorbance of the resulting formazan was measured at 570 nm and 730 nm using a microplate reader.

The cytotoxicity of oxaliplatin and Pt-Luc toward A549-Luc2 cells. A549-Luc2 cells (2.5 x 10^3 per well) were seeded into 96-well plates and incubated at 37 °C for 48 h. The cells were treated with different concentrations of oxaliplatin and **Pt-Luc**, respectively, for 72 h. After that, MTT assays were prepared following the experimental procedures described above.

The cytotoxicity of glutathione (GSH), erastin, N-ethylmaleimide (NEM), and maleimidobutyric acid (MBA) toward A549-Luc2 cells. A549-Luc2 cells (2 x 10^4 per well) were seeded into 96-well plates and cultured for 48 hours. And then undergo different treatments: 1) A549-Luc2 cells were treated with different concentrations of GSH for 24 hours; 2) A549-Luc2 cells were exposed to erastin at a concentration of 100 μM for 5 minutes, followed by two washes with PBS, and subsequently supplemented with 200 μL of fresh medium, allowing for an additional 24-hour growth period, or exposed to erastin at a concentration of 10 μM for 6 hours; 3) A549-Luc2 cells were subjected to treatment with varying concentrations of N-ethylmaleimide (NEM) and maleimidobutyric acid (MBA) for 0.5 hours, respectively. After applying various treatments and respective incubation periods, MTT assays were prepared following the experimental procedures described above.

Cellular bioluminescence assays

Bioluminescence of Pt-Luc. Bioluminescence imaging in all cellular experiments was conducted using a Lumina IVIS Spectrum instrument (PerkinElmer). Prior to assaying, 2 x 10^4 A549-Luc2 cells were passaged and plated in black 96-well plates with clear bottoms (Corning). After 48 hours, the cells were treated with varying concentrations of **Pt-Luc**. The plate was immediately imaged for 180 minutes, with a 30-second exposure time, and segmented with 2-minute delay times. Control experiments were performed by treating cells with 20 μM ligand and imaging under the same conditions.

Bioluminescence of Pt-Luc in BSO-pretreated A549Luc2 cells. To investigate the influence of glutathione (GSH) on the reduction of Pt(IV) complex, A549-Luc2 cells were pretreated with 250 μM of buthionine sulfoximine (BSO) for 24 hours.[4] Subsequently, both A549-Luc2 cells and BSO-pretreated A549-Luc2 cells were incubated with **Pt-Luc** and the ligand, respectively, and imaged accordingly.

Bioluminescence of Pt-Luc in GSH-pretreated A549Luc2 cells. To investigate the effect of exogenous GSH addition on the reduction of the Pt(IV) complex, A549-Luc2 cells were pretreated with 2.5 mM of GSH for 24 hours. Subsequently, both A549-Luc2 cells and GSH-excess A549-Luc2 cells were incubated with **Pt-Luc** and the ligand, respectively, and imaged accordingly.

Bioluminescence of Pt-Luc in erastin-pretreated A549Luc2 cells. To investigate the influence of cysteine on the reduction of Pt(IV) complex, A549-Luc2 cells were exposed to erastin at a concentration of 100 μM for 5 minutes, washed twice with PBS, and then supplemented with 200 μL of fresh medium, allowing for an additional 24-hour growth period, or exposed to erastin at a concentration of 10 μM for 6 hours.[5] Subsequently, both A549-Luc2 cells and erastin-pretreated A549-Luc2 cells were incubated with **Pt-Luc** and the ligand, respectively, and imaged accordingly.

Bioluminescence of Pt-Luc in A549Luc2 cells pretreated with media containing varying levels of L-cystine. To further investigate the impact of cysteine on the reduction of the Pt(IV) complex, the medium of A549-Luc2 cells was replaced with specific media after 24 hours of incubation. The media with different levels of L-cystine were prepared using high glucose, no glutamine, no methionine, and no cystine DMEM as the base. Specifically, the media used were prepared as follows: (1) control medium, containing 200 μM of L-methionine, 150 μM of L-cystine, and 4 mM of L-glutamine; (2) cystine-free medium, comprising 200 μM of L-methionine without L-cystine, and 4 mM of L-glutamine; (3) cystine-excess medium, consisting of 200 μM of L-methionine, 300 μM of L-cystine, and 4 mM of L-glutamine.[6] Subsequently, the pretreated A549-Luc2 cells were incubated with **Pt-Luc** and the ligand, respectively, and imaged accordingly.

Bioluminescence of Pt-Luc in NEM- or MBA-pretreated A549Luc2 cells. To investigate the impact of cellular thiol level on the reduction of Pt(IV) complex, A549-Luc2 cells were subjected to treatment with varying concentrations of N-ethylmaleimide (NEM) and maleimidobutyric acid (MBA) for 0.5 hours, respectively.[7] Subsequently, the pretreated A549-Luc2 cells were incubated with **Pt-Luc** and the ligand, respectively, and imaged accordingly.

Measurement of the reduction products of Pt-Luc in cells by LC-MS

A549-Luc2 cells were seeded into a T-75 flask and cultured for 48 hours until reaching 95% confluency. The cells were then treated with **Pt-Luc** (200 μM, 1% DMF) in an FBS-free medium for 1 hour. Following that, the cells underwent six washes with PBS (12 mL each) and were subsequently incubated in a fresh medium for an additional 3 hours. The cells were collected by trypsinization, washed twice with PBS, and transferred to a 2 mL centrifuge tube. Sonication was used to lyse the cells, with the addition of 50 μL of milli-Q water to the cell pellets. The cell lysate was vortexed for 1 minute and subjected to ultrasonication on ice for 24 cycles of 5 seconds with 5-second intervals. After centrifugation at 12,000 rpm for 10 minutes at 4 ℃, the supernatant was collected. Protein precipitation was achieved by adding 200 μL of methanol to 50 μL of the sample, followed by centrifugation at 14,000 rpm for 2 minutes to remove the precipitated proteins. Finally, 50 μL of supernatant was injected into LC-MS to analyze the reduction products of complex **Pt-Luc**.

Measurement of the GSH and cysteine content

In brief, the cell lysate was prepared following the experimental procedures described above. Subsequently, the supernatant (50 μL) was transferred to a 2.0 mL centrifuge tube and supplemented with glutathione ethyl ester (internal standard, 0.01 mg/ml, 20 μL) and Ellman's reagent (10 mM, 100 μL).[8] After vortex-mixing for 1 minute, 30 μL of 20% 5-sulfosalicylic acid was added to the mixture. Centrifugation (14000 rpm, 10 minutes) was used to remove the precipitated proteins caused by the denaturing and aggregating effects of 5-sulfosalicylic acid, and the resulting supernatant was separated and analyzed by LC-MS. The concentrations of GSH and cysteine were determined relative to a standard curve ranging from 0 to 1000 μM GSH.

Reduction of complex Pt-Luc in high molecular weight (HMW) and low molecular weight (LMW) fractions

In brief, the cell lysate was prepared following the experimental procedures described above. Subsequently, the cell extracts were transferred to Centrifugal Filter Units with a molecular weight cut-off (MWCO) of 3 kDa and centrifuged at 5000 g for 60 minutes at 4 ℃, resulting in the separation of the high molecular weight (HMW) (> 3 kDa) and low molecular weight (LMW) (< 3 kDa) fractions. Both fractions were then diluted with water and 10X PBS to achieve the same volume. The protein and GSH concentrations of these samples were determined by a bicinchoninic acid (BCA) Protein Assay Kit (Beyotime P0011) and a 5,5'-dithiobis(2-nitrobenzoic acid) (DTNB) assay, respectively. MBA was utilized to block thiols in the HMW and LMW fractions. Subsequently, 20 μM of complex **Pt-Luc** was incubated at 37 ℃ in both the MBA-pretreated and untreated HMW and LMW fractions of cell extracts, respectively. Additionally, 2% SDS was employed to induce protein denaturation. At the scheduled time points, the proteins in 50 μL of the sample were precipitated by 200 μL methanol and removed by centrifugation at 14,000 rpm for 5 min. Finally, 100 μL of the supernatant was injected into HPLC to analyze the reduction of complex **Pt-Luc**.

Following the above experimental processes, A549-Luc2 cells were treated with erastin and media containing different levels of L-cystine. Subsequently, the cell lysate was obtained and separated accordingly. The reduction of complex **Pt-Luc** was then assessed in the obtained HMW and LMW fractions.

Measurement of thiol levels

In brief, the HMW and LMW fractions were prepared following the experimental procedures described above. The low molecular weight thiols present in the LMW fraction and the exposed protein thiols in the HMW fraction were measured using 5,5'-dithiobis(2-nitrobenzoic acid) (DTNB) assay.[7a, 9] Total protein thiols were measured after complete denaturation of the proteins with 2% SDS at 37 ℃ for 30 minutes.[10] For this, fractions were diluted (40: 100) with DTNB buffer (10 mM DTNB, 50 mM PB buffer, pH 7.4), incubated for 5 min at room temperature, and the absorbance at 412 nm (A412) was measured using a plate reader. The measurements were compared to a standard curve of 0-1000 μM GSH.

Non-invasive monitoring of the reduction of Pt(IV) prodrugs in animal cancer models

A group of female nude mice were subcutaneously inoculated with ten million A549-Luc2 cells in the right flank. The size of the tumor was carefully monitored. On the fourteenth day post-inoculation, the tumors had grown to a volume of 0.1–0.2 cm^3. The mice were anesthetized in a chamber filled with 2% isofluorane in oxygen and then transferred to the light-tight chamber. The mice received the following treatments: 1) an intraperitoneal (i.p.) injection 1) ligand (50 μM in PBS with 1% DMF, 200 μL per 20 g mouse body weight); 2) **Pt-Luc** (50 μM in PBS with 1% DMF, 200 μL per 20 g mouse body weight). Subsequently, the mice were imaged immediately for 30 minutes using the IVIS spectrum instrument under isoflurane anesthesia (2%), with images captured every minute.

To assess the stability of **Pt-Luc** in blood following intraperitoneal (i.p.) versus intravenous (i.v.) injection, Balb/c nude mice were administered **Pt-Luc** (50 μM, 200 μL per 20 g mouse body weight) via both routes. An equivalent dosage of aminoluciferin was also injected via i.p. and i.v. as a control. Blood samples were collected through submandibular venipuncture and stored in heparin sodium-coated tubes. A volume of 20 μL from each sample was precipitated with 80 μL of 20% 5-sulfosalicylic acid and centrifuged at 14,000 rpm for 10 minutes. Finally, 50 μL of the supernatant was injected into LC-MS to analyze the stability of the **Pt-Luc** complex.

To assess the stability of **Pt-Luc** in whole blood from mice, blood samples were collected via submandibular venipuncture and stored in heparin sodium-coated tubes. The Pt(IV) complex **Pt-Luc** (50 μM, 1% DMF) was incubated in the blood at 37 °C under light protection. At designated time points, 20 μL of the sample was precipitated with 80 μL of 20% 5-sulfosalicylic acid and centrifuged at 14,000 rpm for 10 minutes. Finally, 50 μL of the supernatant was injected into HPLC to analyze the stability of the **Pt-Luc** complex.

To investigate the direct effect of intraperitoneally injected NEM on active protein thiols in tumors, Balb/c nude mice bearing A549-Luc2 tumor xenografts received an intraperitoneal injection of NEM (100 μM in PBS, 200 μL per 20 g mouse body weight). The control group was administered an equivalent volume of PBS. After 30 minutes, tumors were harvested, snap-frozen in liquid nitrogen, and ground to a fine powder using a glass homogenizer. Subsequently, 500 μL of Radioimmunoprecipitation assay buffer (RIPA buffer; 25 mM Tris•HCl pH 7.6, 150 mM NaCl, 1% NP-40, 1% sodium deoxycholate, 0.1% SDS) was added to the powdered tumors, and the mixture was thoroughly homogenized. The solution was transferred to a 2 mL tube and vortexed for 30 minutes, with vortexing occurring every minute. To ensure complete cell lysis, the solution underwent ultrasonication on ice for 24 cycles of 5 seconds each, with 5-second intervals. Following centrifugation at 12,000 rpm for 10 minutes at 4 °C, the supernatant was collected. Protein concentrations were determined using a Bicinchoninic Acid (BCA) Protein Assay Kit (Beyotime P0011), and the protein concentration in each group was adjusted to 4 mg/mL. Thiol levels in each sample were measured using a 5,5'-dithiobis(2-nitrobenzoic acid) (DTNB) assay.

To investigate the role of protein thiols in the reduction of Pt(IV) complexes in vivo, mice in groups 1 and 3 were administered an i.p. injection of the thiol-blocking agent NEM (100 μM in PBS buffer, 200 μL per 20 g mouse body weight). In contrast, mice in groups 2 and 4 received PBS only. After a 30-minute interval, mice in groups 1 and 2 were given an i.p. injection of **Pt-Luc** (50 μM in PBS with 1% DMF, 200 μL per 20 g mouse body weight), while those in groups 3 and 4 were injected with aminoluciferin (50 μM in PBS with 1% DMF, 200 μL per 20 g mouse body weight). The mice were imaged immediately for 30 minutes using the IVIS spectrum instrument under isoflurane anesthesia (2%), with images captured every minute.

Animal ethics

The mice, all purchased from the City University of Hong Kong, were reared in an environment free from pathogens. Every experiment involving animals was executed in strict accordance with the City University of Hong Kong's Guidelines for the Care and Use of Laboratory Animals. These experiments received the necessary approval from the Animal Research Ethics Sub-Committee of the City University of Hong Kong, under the approval number AN-STA-00000308.

Data Availability Statement

The data that support the findings of this study are available from the corresponding author upon reasonable request.

Results and Discussion

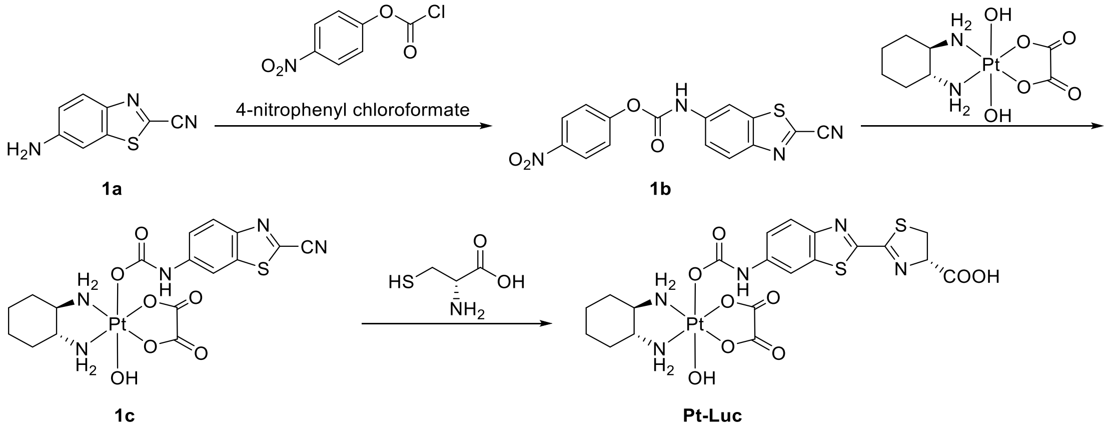

Scheme S1. Synthetic route for bioluminescent probe **Pt-Luc**.

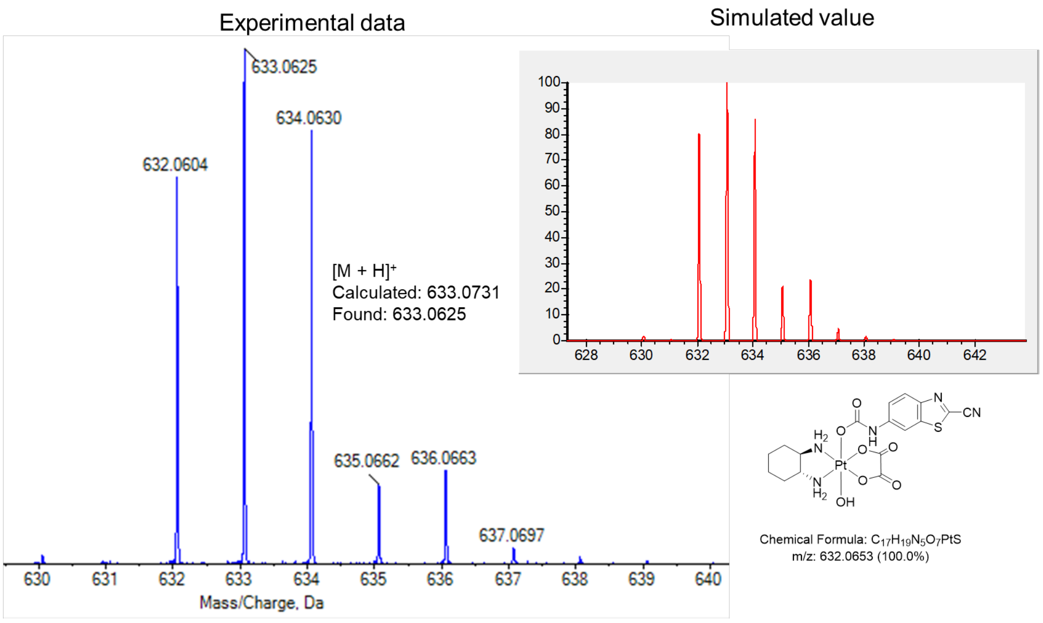

Figure S1. ESI-HRMS spectrum of complex **1c**.

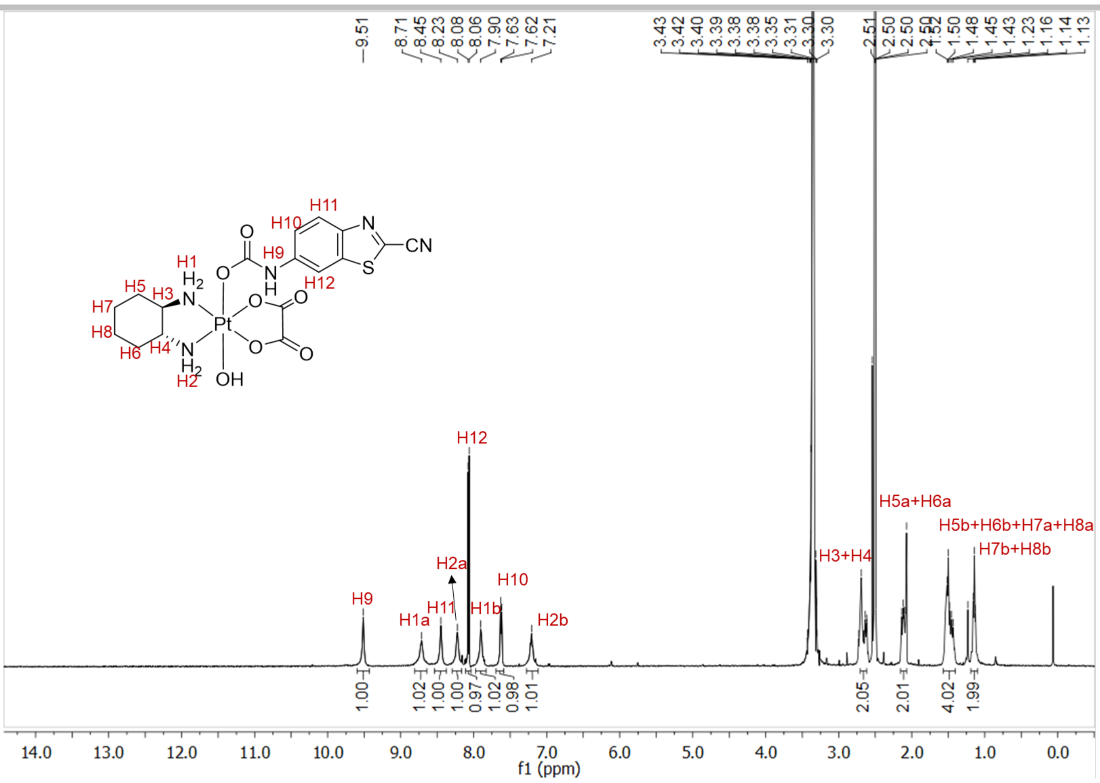

Figure S2. ^{1}H NMR spectrum of complex **1c** in DMSO-*d*6.

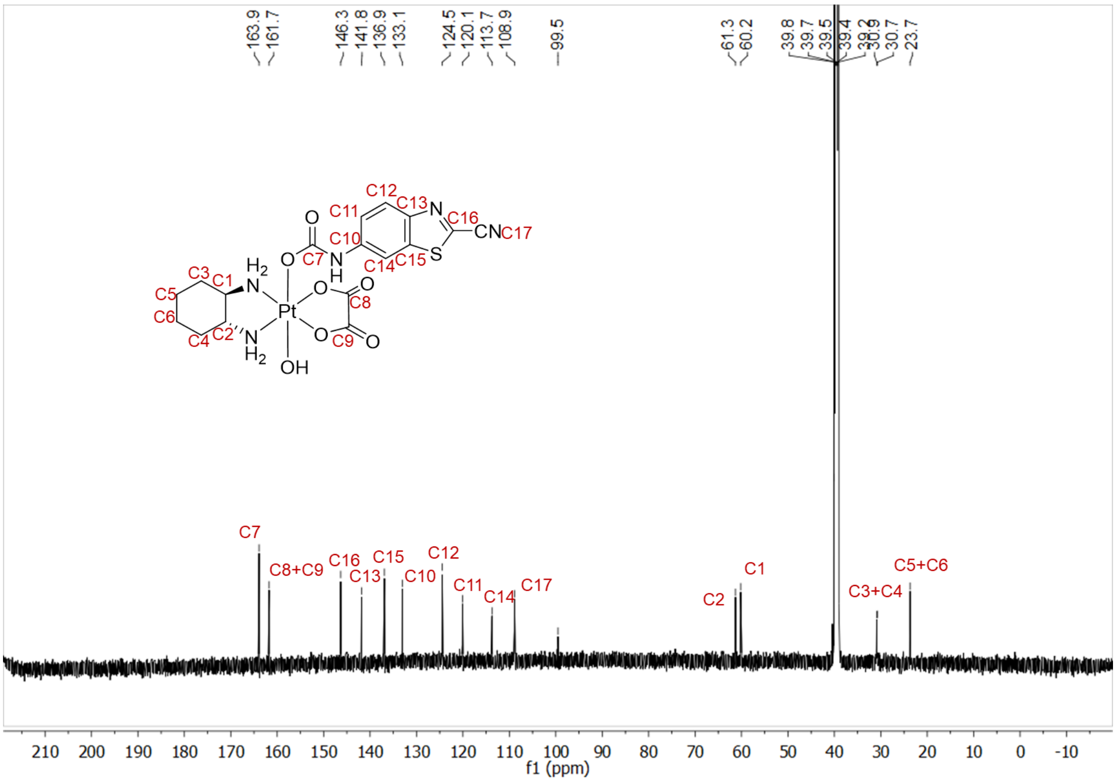

Figure S3. ^{13}C NMR spectrum of complex **1c** in DMSO-*d*6.

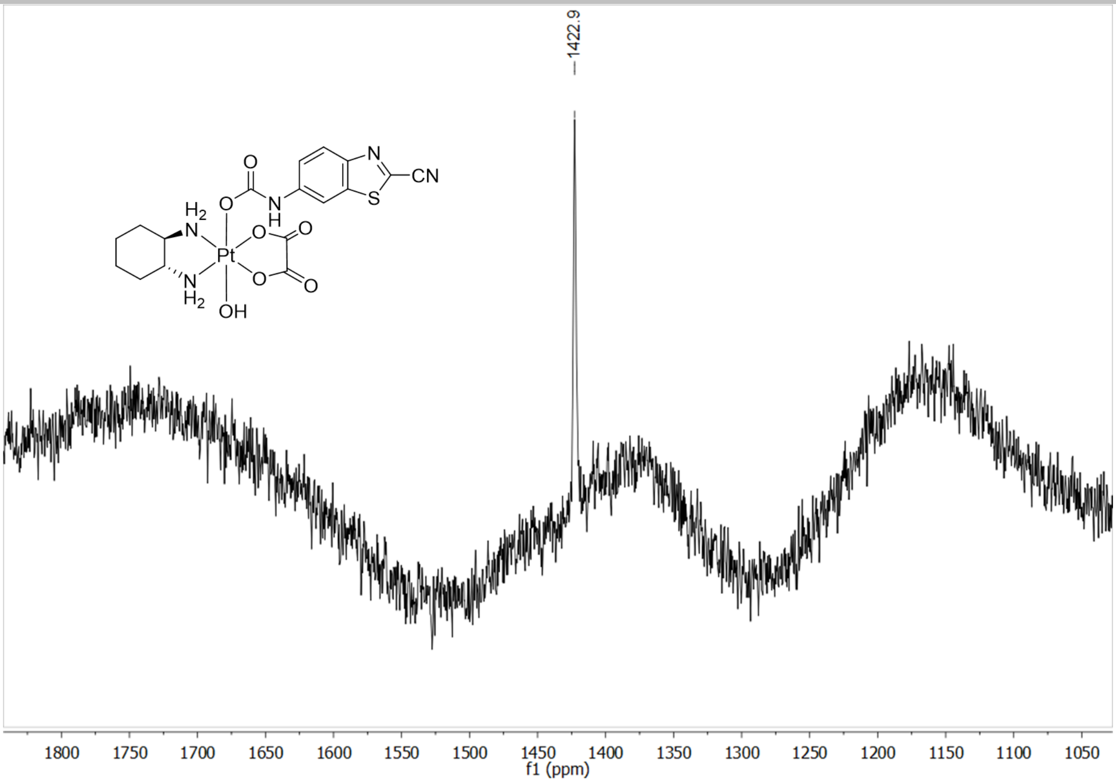

Figure S4. ^{195}Pt NMR spectrum of complex **1c** in DMSO-d_6.

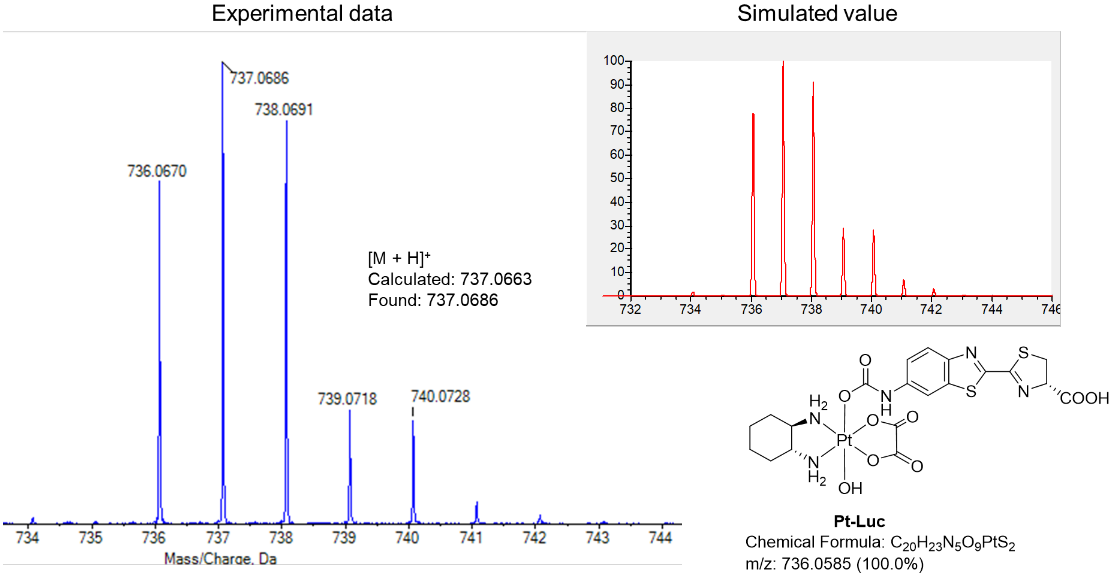

Figure S5. ESI-HRMS spectrum of complex **Pt-Luc**.

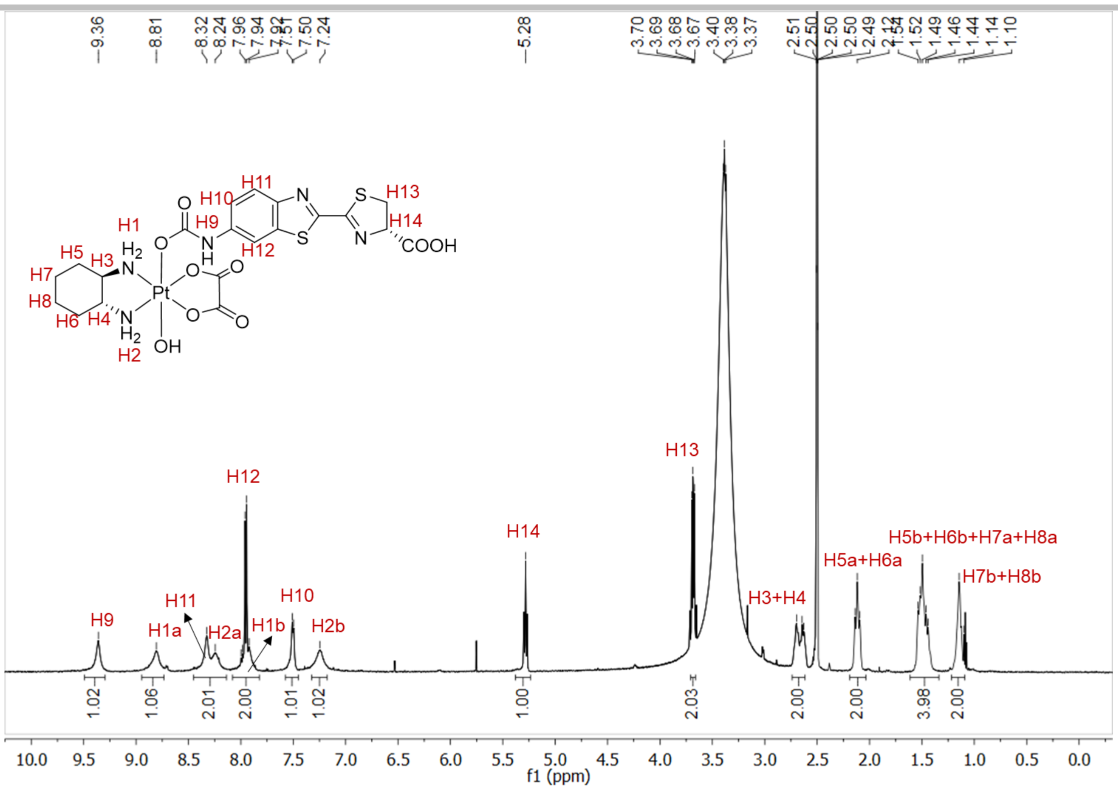

Figure S6. ^{1}H NMR spectrum of complex **Pt-Luc** in DMSO-*d*6.

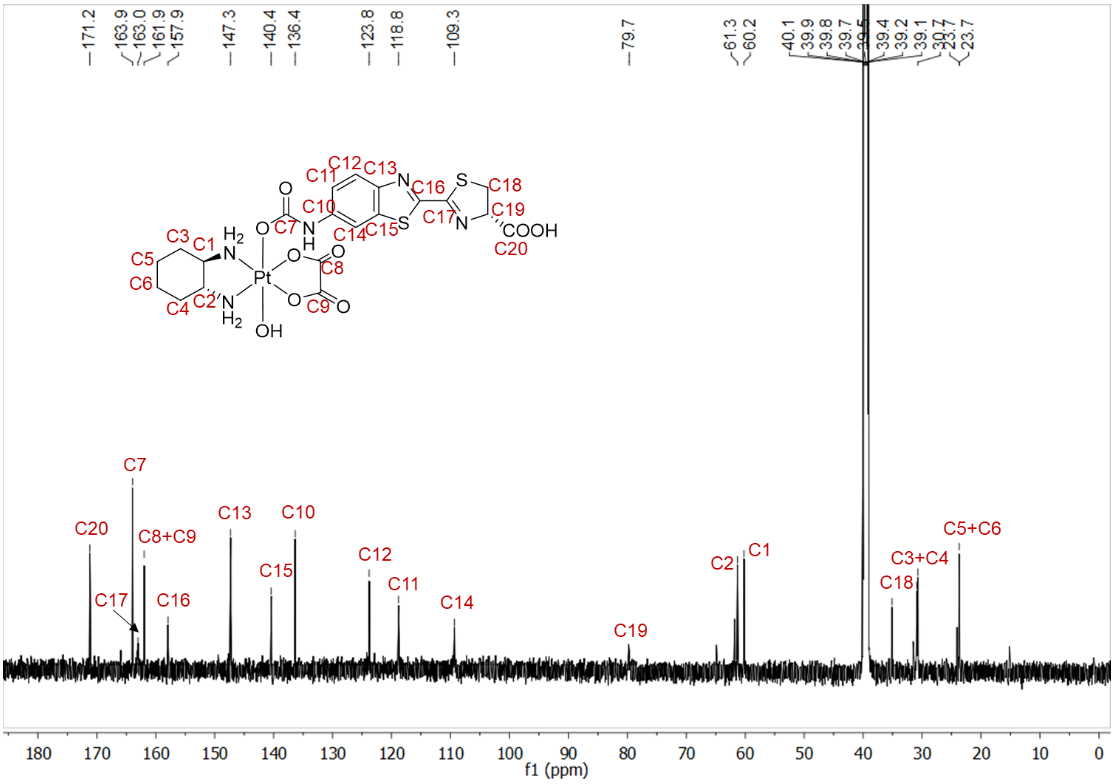

Figure S7. ^{13}C NMR spectrum of complex **Pt-Luc** in DMSO-*d*6.

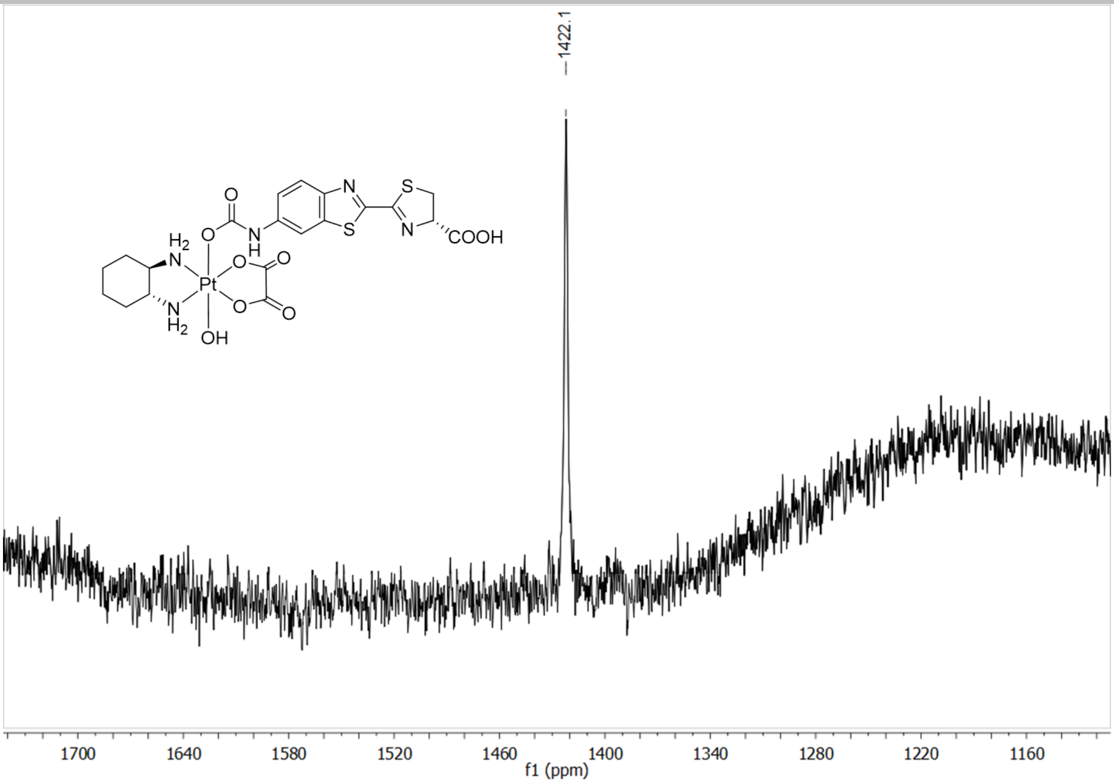

Figure S8. ^{195}Pt NMR spectrum of complex **Pt-Luc** in DMSO-d_6.

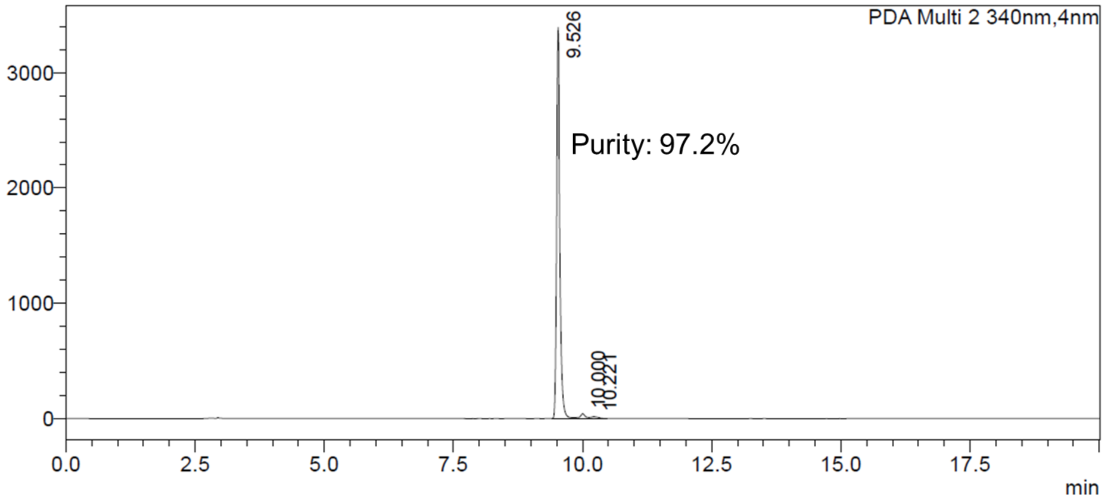

Figure S9. HPLC chromatogram for the purity test of **Pt-Luc**.

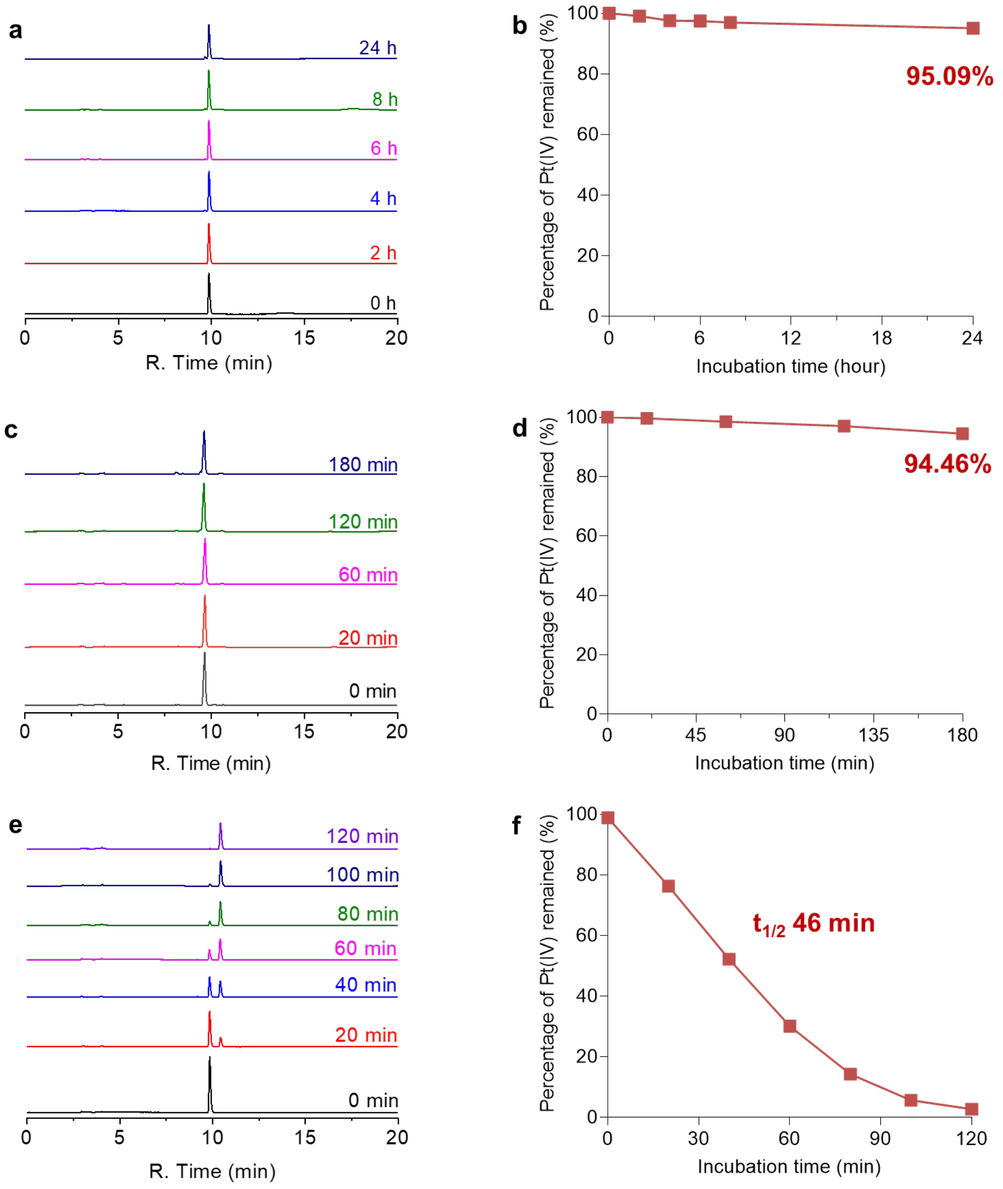

Figure S10. The stability and reduction tests of the bioluminescent Pt(IV) probe. (a) The HPLC chromatogram of **Pt-Luc** in PB buffer (50 mM, pH 7.4) at 37 ℃ for 24 h. (b) The percentage of remaining Pt(IV) complex **Pt-Luc** during the incubation in PB buffer (50 mM, pH 7.4) at 37 ℃ for 24 h. (c) HPLC chromatogram of **Pt-Luc** incubating in F-12K medium at 37 ℃. (d) The percentage of remaining **Pt-Luc** during the incubation in F-12K medium at 37 ℃. (e) HPLC chromatogram of **Pt-Luc** in PB buffer (50 mM, pH 7.4) at 37 ℃ containing 2 mM sodium ascorbate. (f) The percentage of remaining Pt(IV) complex **Pt-Luc** in a PB buffer (50 mM, pH 7.4) at 37 ℃ with 2 mM sodium ascorbate.

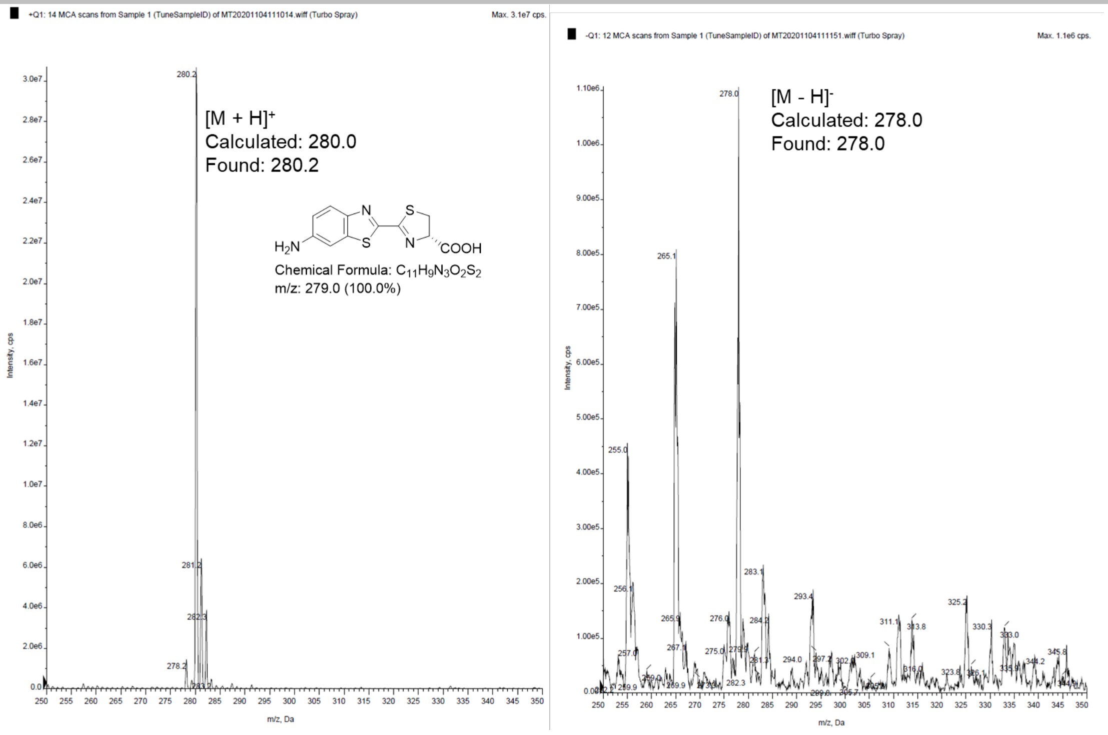

Figure S11. The ESI-Mass spectrum of reduction product with a retention time of 10.8 min.

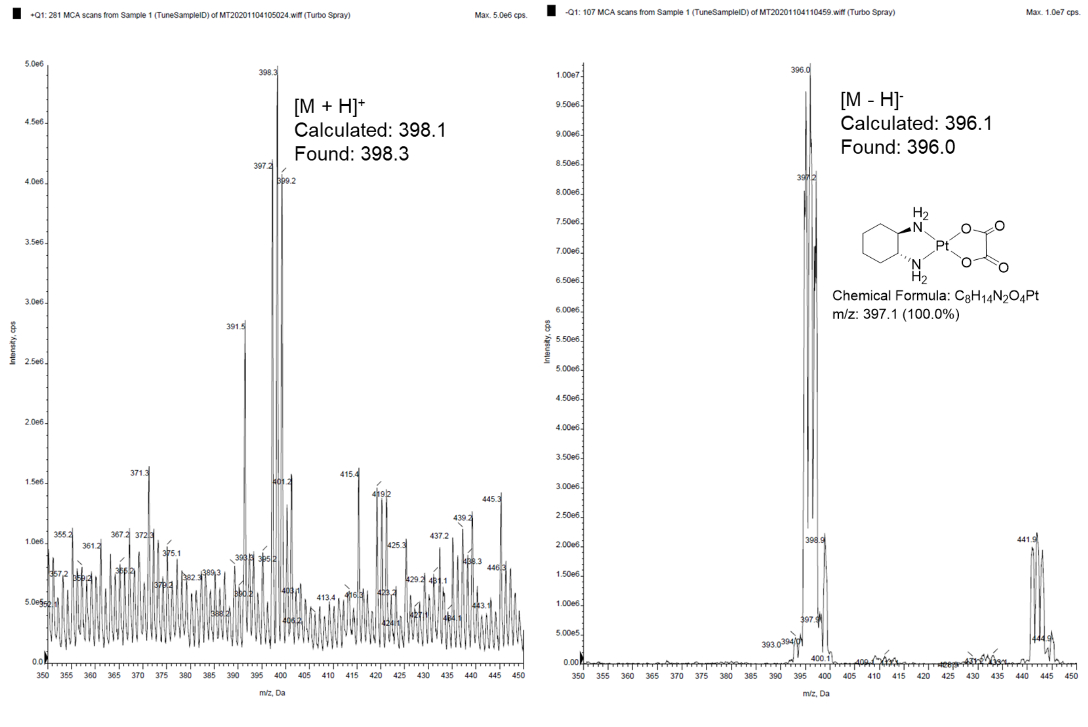

Figure S12. The ESI-Mass spectrum of reduction product with a retention time of 6.8 min.

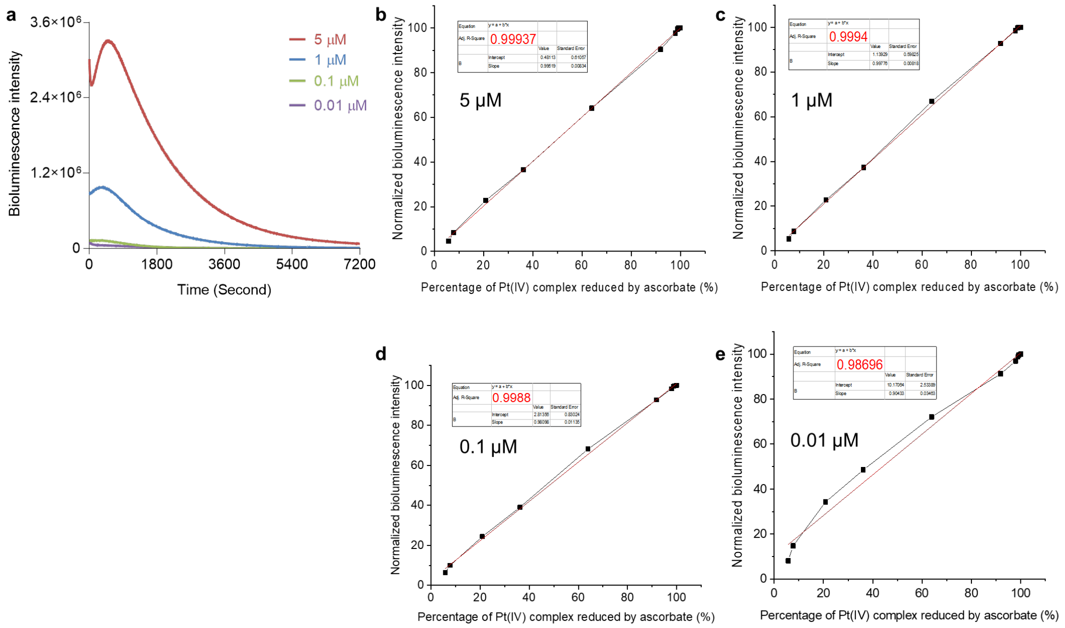

Figure S13. The detection limit of Pt(IV) complex **Pt-Luc** in a buffer. (a) Time-dependent bioluminescent signal generation of **Pt-Luc** in a mixture solution. 100 µL of a solution of luciferase (100 µg/ml) in 50 mM Tris buffer at pH 7.4 containing 10 mM Mg^{2+} ($MgCl_2$), 0.1 mM Zn^{2+} ($ZnCl_2$), and 2 mM ATP was mixed with 100 µL of different concentrations of **Pt-Luc** (5 µM, 1 µM, 0.1 µM, 0.01 µM). The resulting mixture was shaken for 10 s and read at 37 ℃ for 2 h. The bioluminescence data was plotted against the percentage of Pt(IV) complex reduced, as measured by HPLC. (b-e) Line graphs showing the relationship between the increment of bioluminescence intensity of 5 µM, 1 µM, 0.1 µM, and 0.01 µM of **Pt-Luc** and the percentage of Pt(IV) complex reduced.

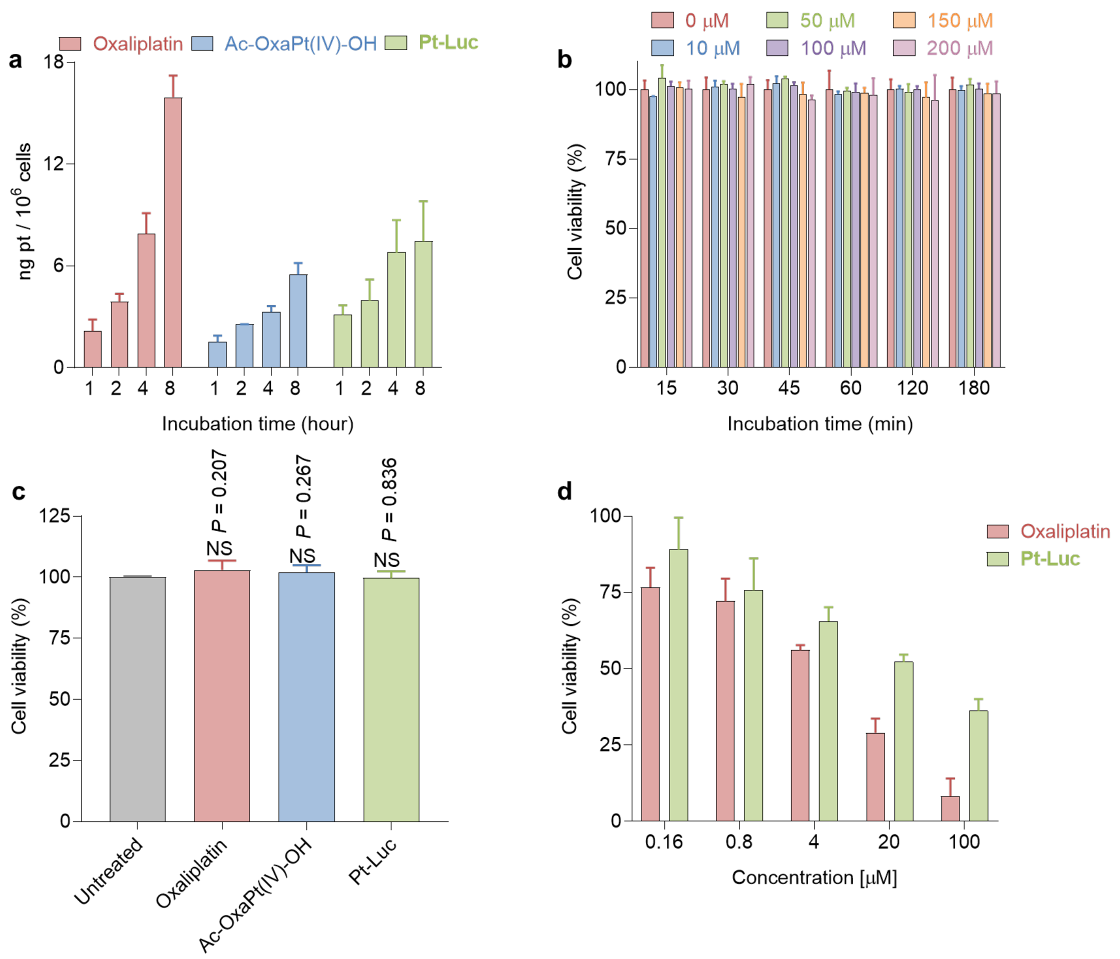

Figure S14. (a) Cellular accumulation of oxaliplatin, *t*-[Pt(DACH)(ox)(OH)($OOCCH_3$)] (Ac-OxaPt(IV)-OH), and **Pt-Luc** in A549-Luc2 cells. (b) The influence of **Pt-Luc** on cell viability was determined using the MTT assay across three hours. Data are presented as mean ± SD, n = 6. (c) Cell viability of A549-Luc2 cells treated with 200 μM oxaliplatin, *t*-[Pt(DACH)(ox)(OH)($OOCCH_3$)] (Ac-OxaPt(IV)-OH), or **Pt-Luc** for three hours was assessed using the MTT assay. Data are expressed as mean ± SD, n = 4. The results showed that neither Ac-OxaPt(IV)-OH nor oxaliplatin exhibited toxicity at 200 μM after 3 hours. (D) The cell viability of **Pt-Luc** and oxaliplatin in A549-Luc2 cells following a 72-hour treatment. The findings demonstrated that **Pt-Luc** induced significant toxicity after 72 hours, with an IC_{50} of 22.8 ± 5.8 μM, compared to 2.6 ± 1.0 μM for oxaliplatin.

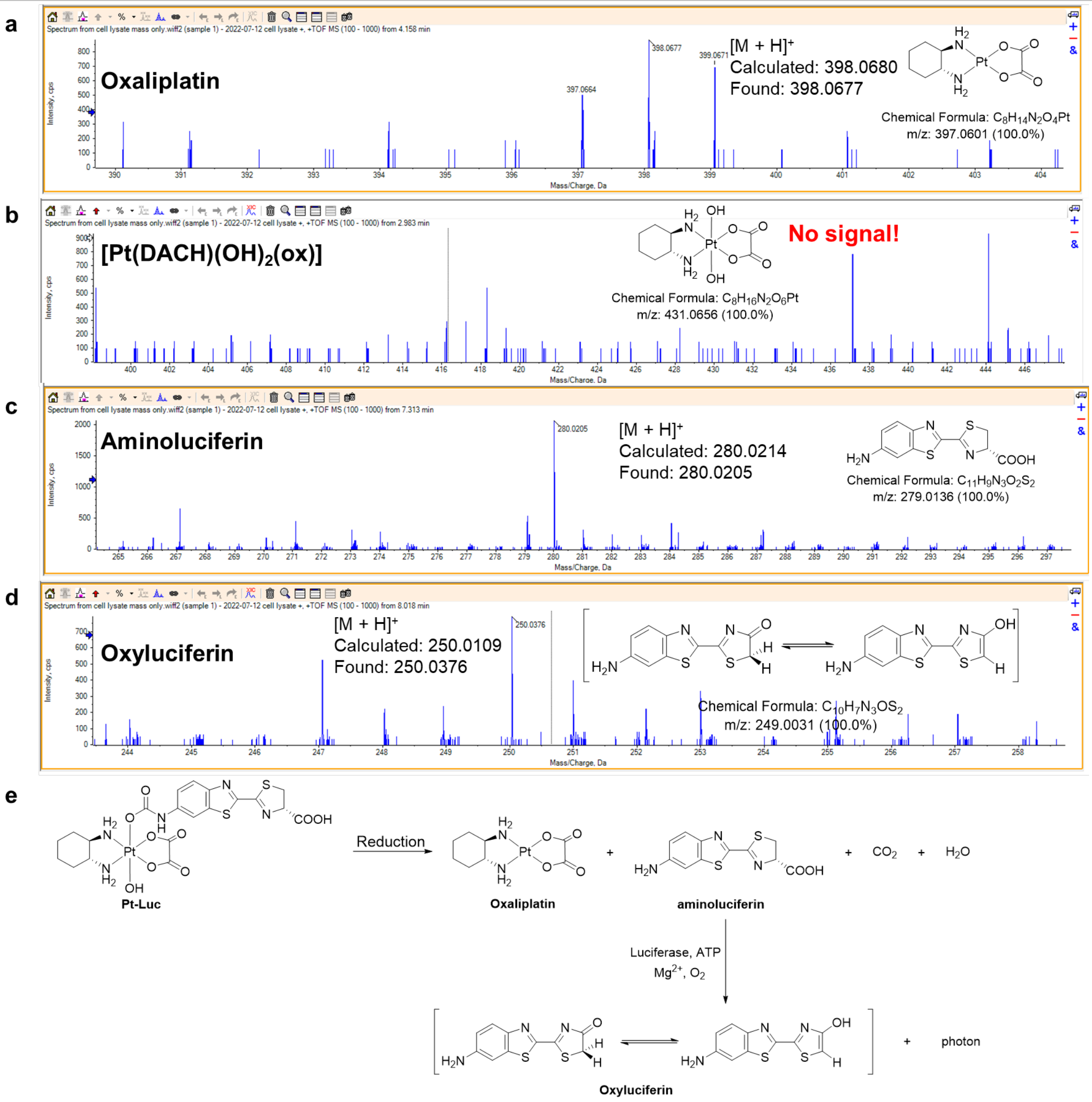

Figure S15. The reduction products of **Pt-Luc** in A549-Luc2 cells. The cells were incubated with **Pt-Luc** (200 μM, 1% DMF) in an FBS-free medium for 1 h. The cells were then collected and lysed. The supernatant was injected into LC-MS to analyze the reduction products of complex **Pt-Luc**. The extracted complexes are (a) oxaliplatin, (b) [Pt(DACH)(OH)2(ox)], (c) aminoluciferin, and (d) oxyluciferin. (e) The proposed reduction pathway of **Pt-Luc** and oxidized pathway of aminoluciferin in A549-Luc2 cells.

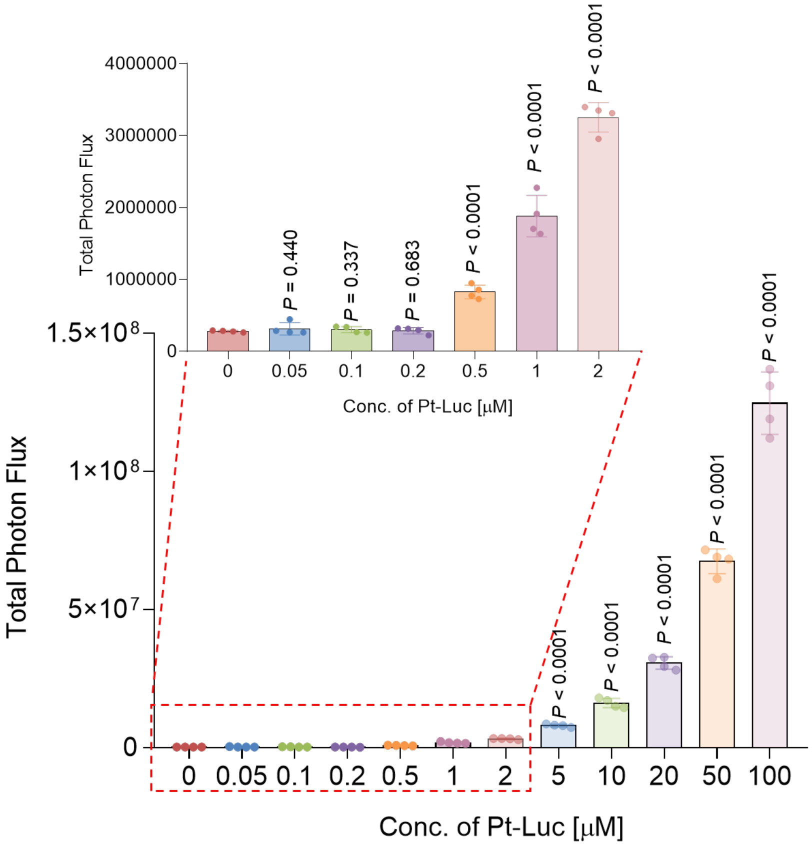

Figure S16. The detection limit of Pt(IV) complex **Pt-Luc** in live cells. The total photon flux, integrated over 3 h, was derived from A549-Luc2 cells treated with different concentrations of **Pt-Luc**. Statistical analyses were performed using a two-tailed Student's test, and error bars represent ± SD (n = 4).

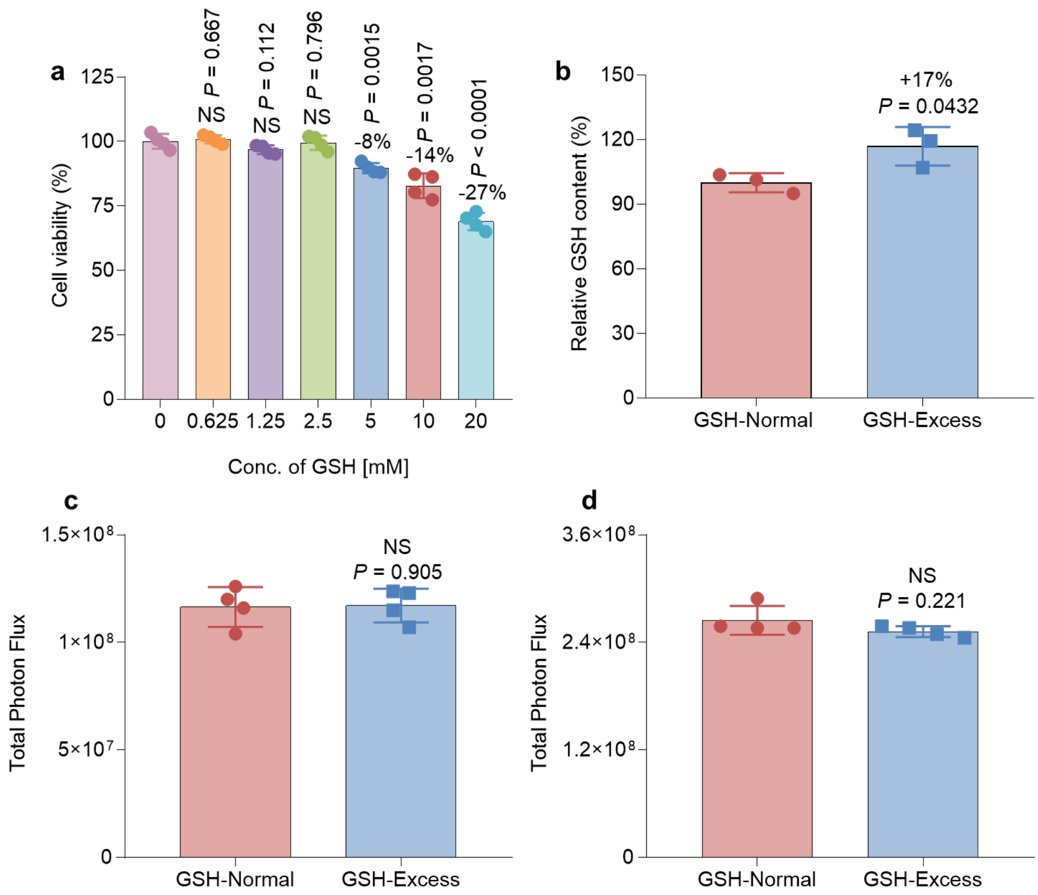

Figure S17. The application of **Pt-Luc** to investigate the role of exogenous GSH addition in the reduction of Pt(IV) complexes in live cells. (a) Cell viability of A549-Luc2 cells treated with various concentrations of GSH for 24 hours. This experiment aimed to identify the maximum concentration of GSH that can be added without adversely affecting cell viability. (b) The GSH contents from A549-Luc2 cells were measured after preincubation of the cells with or without 2.5 mM GSH for 24 h. Bioluminescent signals of **Pt-Luc** (c) and aminoluciferin (d) from the GSH-normal and GSH-excess A549-Luc2 cells. Total photon flux was integrated over 3 h. Statistical analyses were performed using a two-tailed Student's test, and error bars represent ± SD (n = 4).

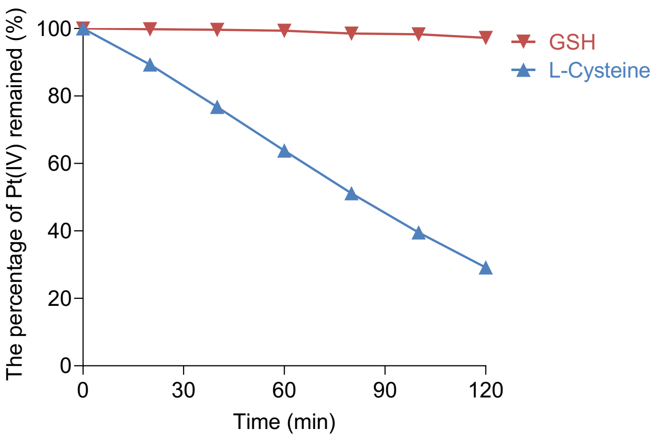

Figure S18. The percentage of remaining Pt(IV) complex **Pt-Luc** during the incubation in a PB buffer (50 mM, pH 7.4) at 37 °C containing 2 mM GSH and L-cysteine, respectively.

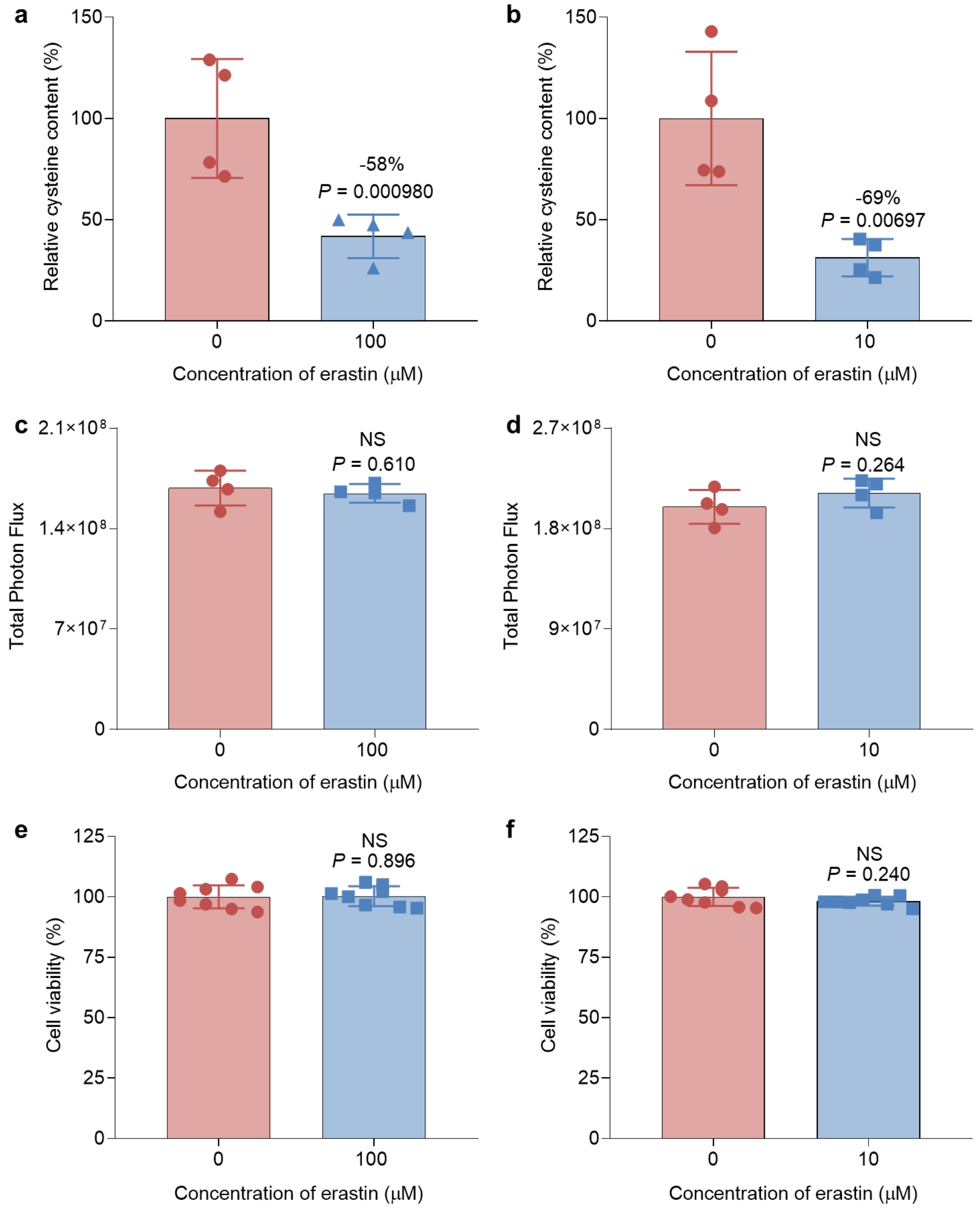

Figure S19. The application of **Pt-Luc** to investigate the role cysteine plays in the reduction of Pt(IV) complexes in live cells. The cysteine content from A549-Luc2 cells was measured after preincubation of the cells with (a) 100 μM erastin for 5 min, followed by washing and incubation for an additional 24 h, or (b) 10 μM erastin for 6 h. The influence of (c) 100 μM erastin for 5 min and (d) 10 μM erastin for 6 h on the activity of luciferase was determined by measuring bioluminescent signals of aminoluciferin from A549-Luc2 cells. The influence of (e) 100 μM erastin for 5 min and (f) 10 μM erastin for 6 h on cell viability was determined using the MTT assay.

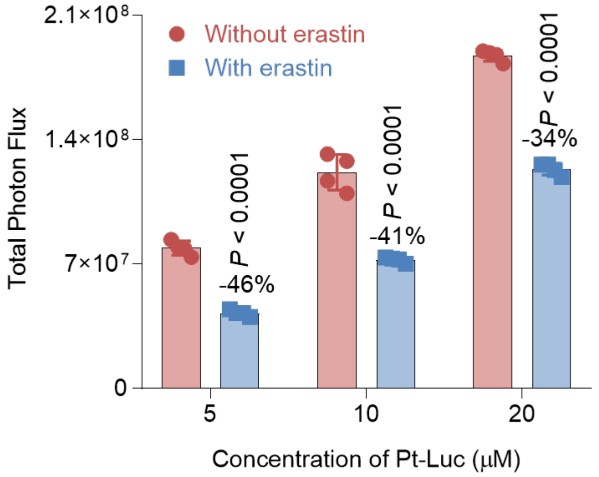

Figure S20. Total photon flux from A549-Luc2 cells after preincubation of the cells with 10 μM erastin for 6 h, washing, and addition of medium containing different concentrations of **Pt-Luc**. The total photon flux was integrated over 3 h. Statistical analyses were performed using a two-tailed Student's test, and error bars represent ± SD (n = 4).

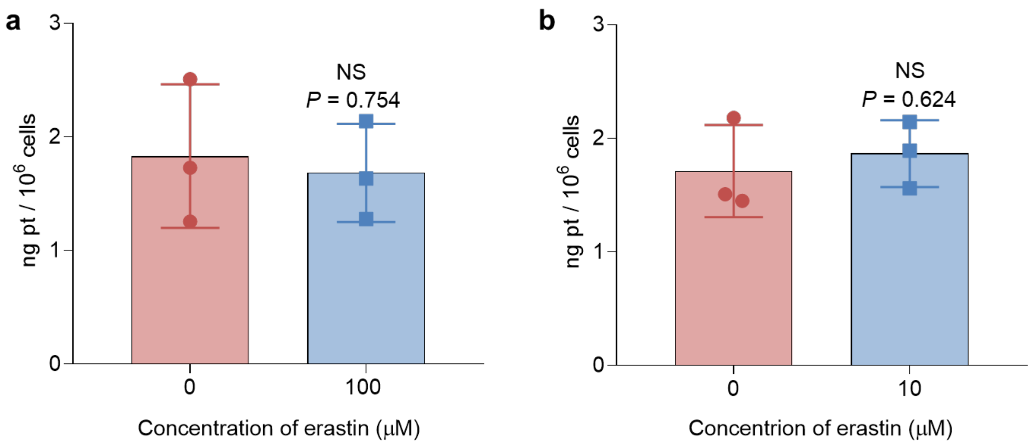

Figure S21. The cellular accumulation of **Pt-Luc** in A549-Luc2 cells was measured after preincubation of the cells with or without (a) 100 μM erastin for 5 min, followed by washing and a 24 h incubation, or (b) 10 μM erastin for 6 h.

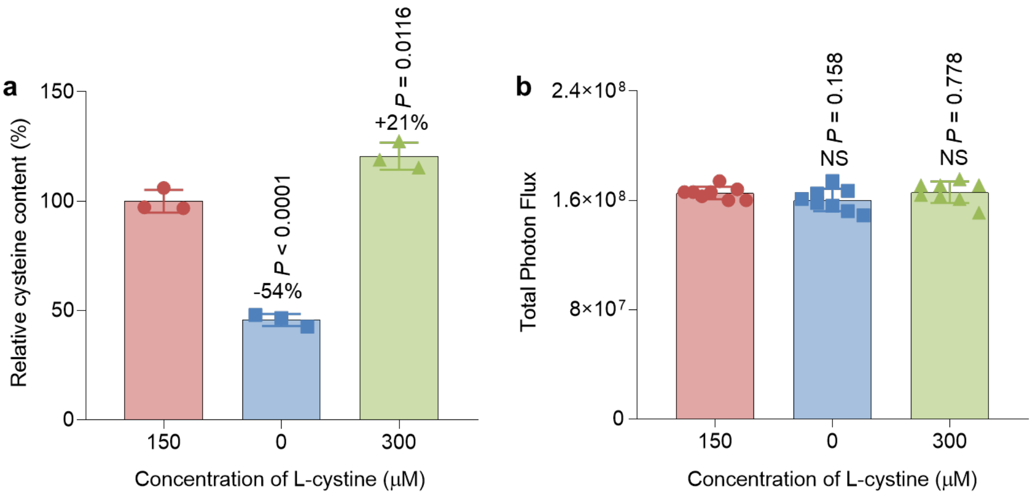

Figure S22. (a) The cysteine contents in A549-Luc2 cells were measured after preincubation of the cells with L-cystine normal, L-cystine free, and L-cystine excess medium, respectively, for 24 h. Data are presented as mean ± SD, n = 3. (b) Total photon flux from A549-Luc2 cells incubated with 20 μM of aminoluciferin was measured after preincubation of the cells with L-cystine normal, L-cystine free, and L-cystine excess medium, respectively, for 24 h. Data are presented as mean ± SD, n = 8.

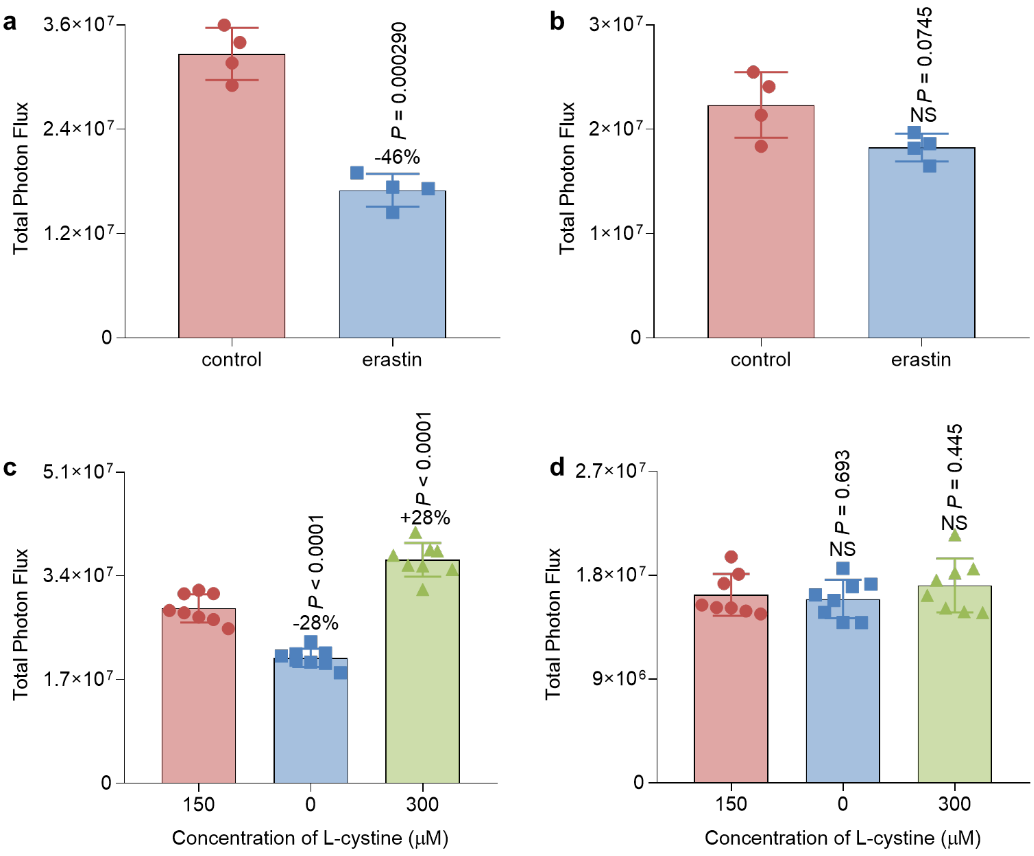

Figure S23. The application of **Pt-Luc** to investigate the role cysteine plays in the reduction of Pt(IV) complexes in MDA-MB-231-Luc cells. Total photon flux from MDA-MB-231-Luc cells incubated with either (a) 20 μM of **Pt-Luc** or (b) 20 μM of aminoluciferin was measured after preincubation of the cells with 100 μM erastin for 5 min, followed by washing and incubation for an additional 24 h. Data are presented as mean ± SD, n = 4. Total photon flux from MDA-MB-231-Luc cells incubated with either (c) 20 μM of **Pt-Luc** or (d) 20 μM of aminoluciferin was measured after preincubation of the cells with L-cystine normal, L-cystine free, and L-cystine excess medium, respectively, for 24 h. Data are presented as mean ± SD, n = 8.

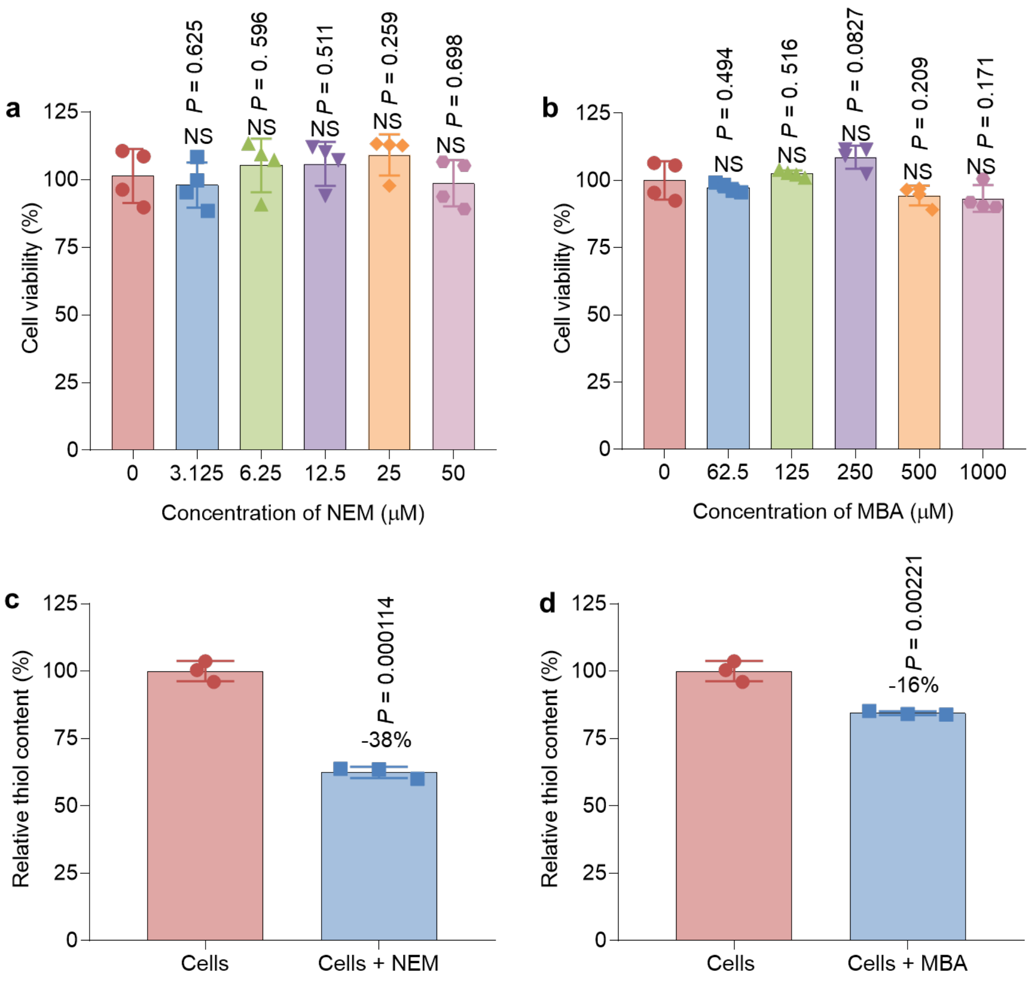

Figure S24. (a) The influence of N-Ethylmaleimide (NEM) on cell viability was determined using the MTT assay. (b) The influence of maleimidobutyric acid (MBA) on cell viability was determined using the MTT assay. The results showed that NEM and MBA were non-toxic to cells at these treated concentrations. The concentration of thiol levels was measured in the whole cell lysate of the A549-Luc2 cells that were preincubated with (c) 50 μM of NEM or (d) 1 mM MBA, respectively, for 0.5 h.

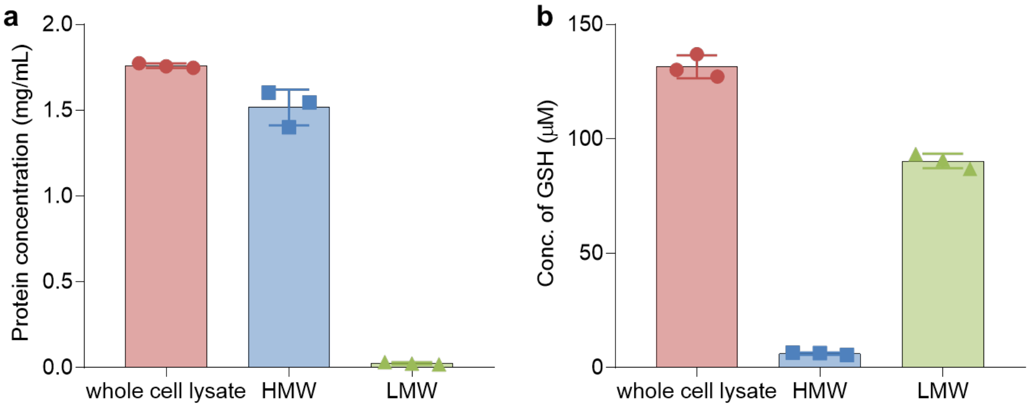

Figure S25. The extract of A549-Luc2 cells was separated using Centrifugal Filter Units with a 3 kDa molecule weight cut-off (MWCO) to yield the high molecular weight (HMW, >3 kDa) and the low molecular weight (LMW, <3 kDa) portions. The protein (a) and GSH (b) concentrations in each portion were determined by BCA and DTNB assays, respectively, which indicate the high separation efficiency.

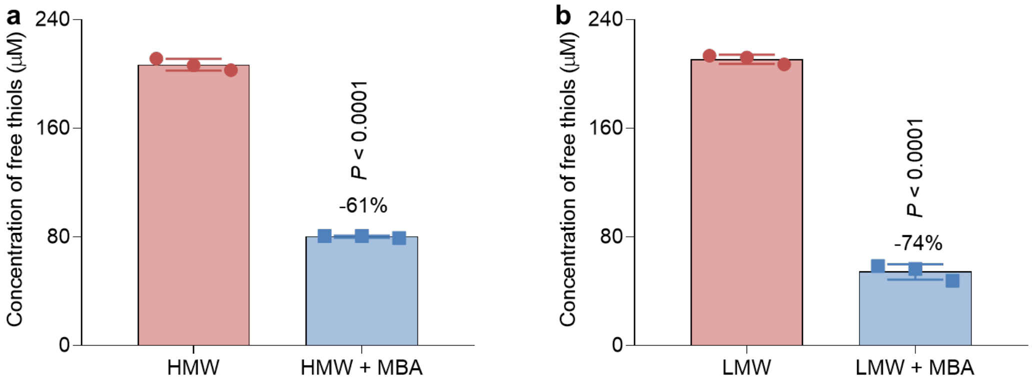

Figure S26. (a) The concentration of free thiols was measured in the high molecular weight (HMW) fraction, with or without treatment with maleimidobutyric acid (MBA). (b) The concentration of free thiols was measured in the low molecular weight (LMW) fraction, with or without treatment with MBA. The initial concentrations of thiols in the HMW and LMW fractions were adjusted to the same level.

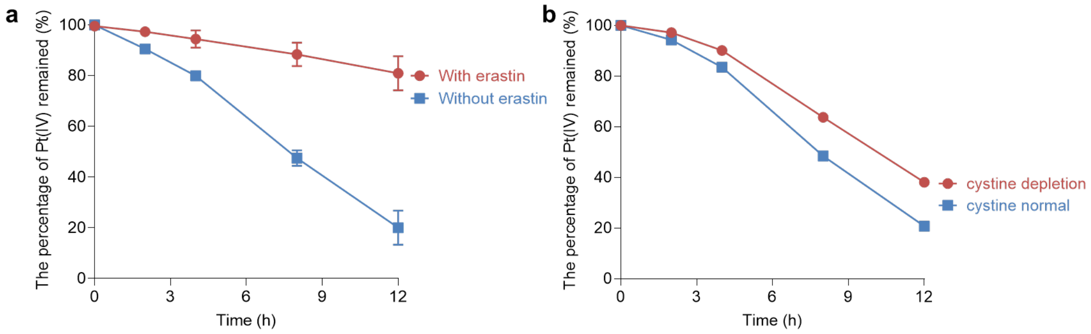

Figure S27. The reduction of **Pt-Luc** in high molecular weight (HMW) portions, separated from the A549-Luc2 cell extracts, was measured by HPLC. (a) The percentage of remaining Pt(IV) complex **Pt-Luc** was measured in the HMW portion of A549-Luc2 cell extracts. The A549-Luc2 cells were pretreated with or without 100 μM erastin for 5 min, then washed and incubated for another 24 h. (b) The percentage of remaining Pt(IV) complex was measured in the HMW portion of A549-Luc2 cell extracts. The cells were pretreated with L-cystine normal and L-cystine free medium, respectively, for 24 h.

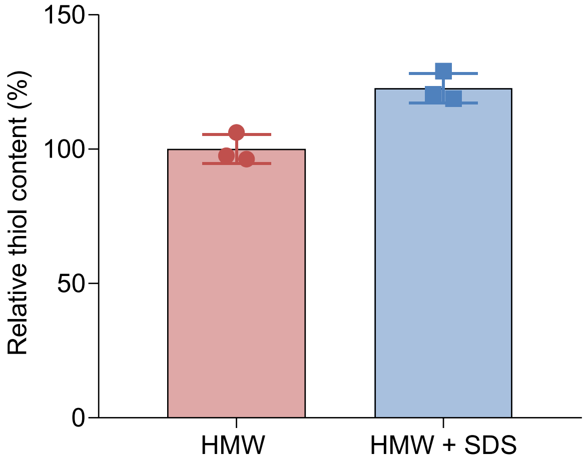

Figure S28. The concentration of thiols was measured in the high molecular weight (HMW) portion of the A549-Luc2 cell extract that was pretreated with or without sodium dodecyl sulfate (SDS). When proteins are denatured using SDS, the thiol levels can appear to increase due to the unfolding of the protein structure, exposing buried thiol groups that were previously inaccessible.

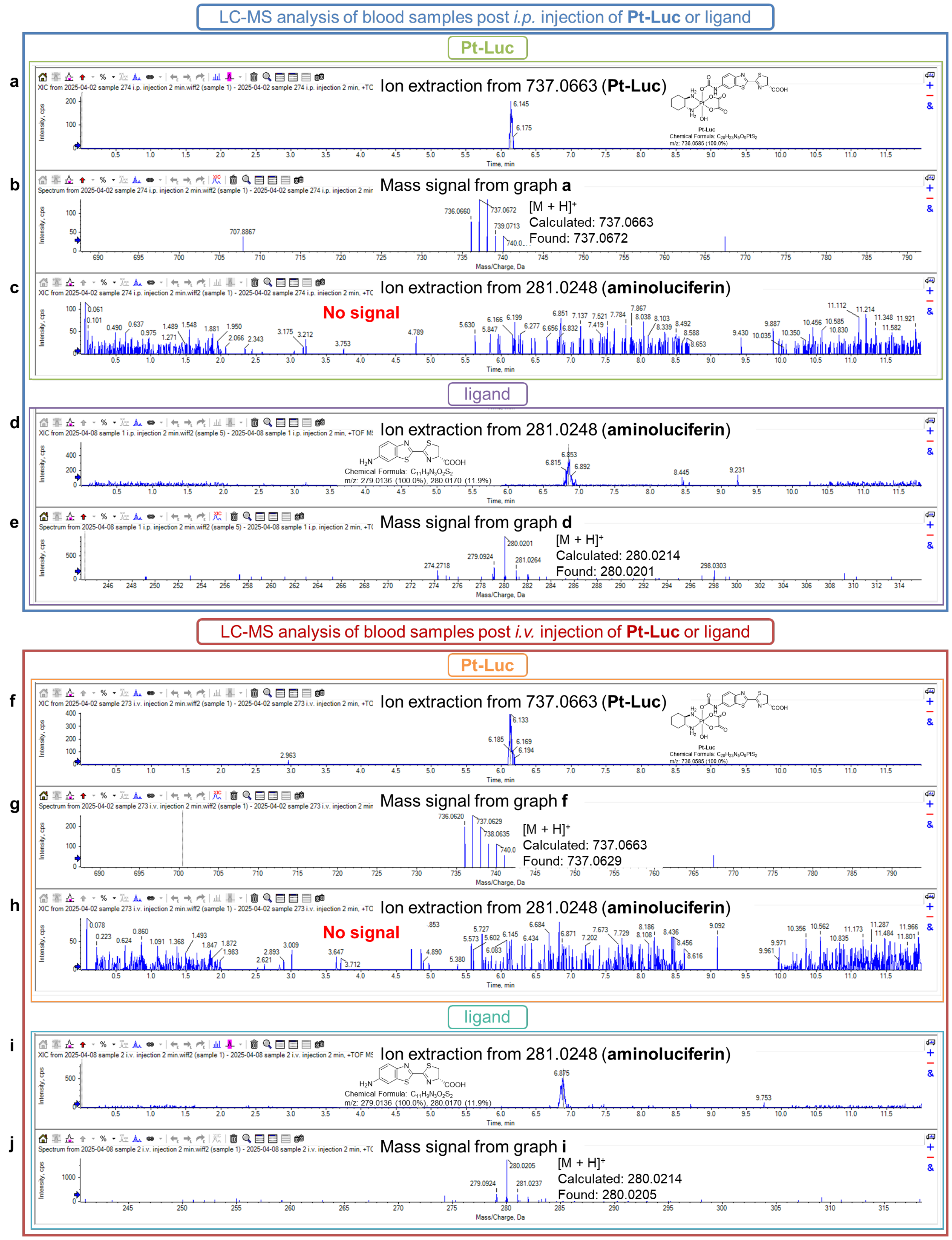

Figure S29. Stability assessment of **Pt-Luc** in blood following intraperitoneal (i.p.) versus intravenous (i.v.) injection in BALB/c nude mice. (a-e) LC-MS analysis of blood samples post i.p. injection of **Pt-Luc** or ligand: (a-c) LC-MS analysis of blood 2 min post i.p. injection of **Pt-Luc** (50 µM, 200 µL per 20 g mouse body weight): (a) Extracted ion chromatogram for m/z 737.0663 ($[M+H]^+$ ion of **Pt-Luc**). (b) High-resolution mass spectrum (HRMS) of the peak in (a), confirming alignment with the theoretical m/z of **Pt-Luc** ($[M+H]^+$, 737.0663). (c) Extracted ion chromatogram for m/z 281.0248 ($[M+H]^+$ ion of aminoluciferin); no detectable signal suggests minimal degradation of **Pt-Luc** during peritoneal-to-blood transport. (d-e) LC-MS analysis of blood 2 min post i.p. injection of ligand aminoluciferin (50 µM, 200 µL per 20 g mouse body weight): (d) Extracted ion chromatogram for m/z 281.0248 (the secondary $[M+H]^+$ ion for aminoluciferin, which was chosen to minimize interference). (e) High-resolution mass spectrum from panel (d), confirming alignment with the theoretical m/z of aminoluciferin $[M+H]^+$ (280.0124). (f-j) LC-MS analysis of blood samples post i.v. injection of **Pt-**

Luc or Ligand: (f-h) LC-MS analysis of blood 2 min post i.v. injection of **Pt-Luc** (50 μM, 200 μL per 20 g mouse body weight): (f) Extracted ion chromatogram for m/z 737.0663 ($[M+H]^+$ ion of **Pt-Luc**). (g) High-resolution mass spectrum (HRMS) of the peak in (f), confirming alignment with the theoretical m/z of **Pt-Luc** ($[M+H]^+$, 737.0663). (h) Extracted ion chromatogram for m/z 281.0248; absence of aminoluciferin signal (consistent with i.p. results) implies the stability of **Pt-Luc** in blood under both administration routes. (i-j) LC-MS analysis of blood 2 min post i.v. injection of aminoluciferin (50 μM, 200 μL per 20 g mouse body weight): (i) Extracted ion chromatogram for m/z 281.0248. (j) High-resolution mass spectrum from panel (i), confirming alignment with the theoretical m/z of aminoluciferin $[M+H]^+$ (280.0124).

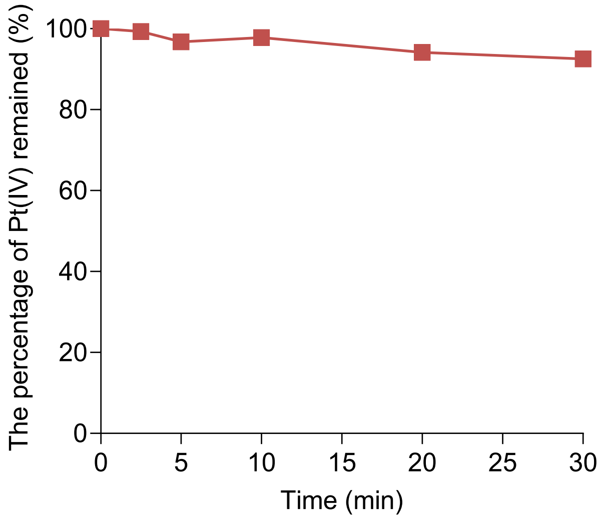

Figure S30. The percentage of remaining Pt(IV) complex **Pt-Luc** during the incubation in whole blood at 37 °C.

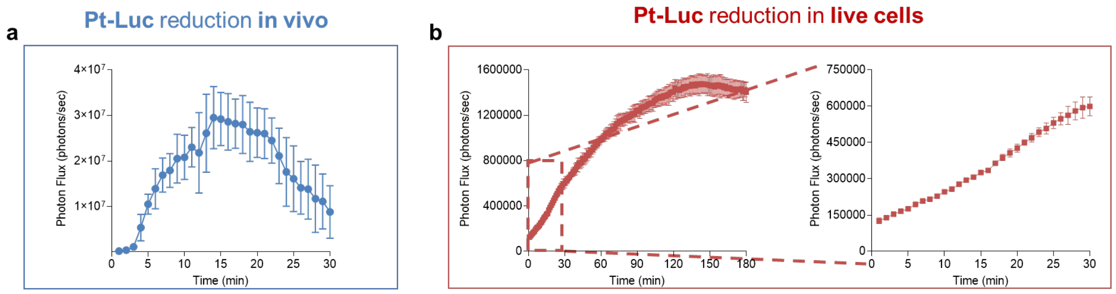

Figure S31. (a) Time-dependent bioluminescent signal generation of **Pt-Luc** from A549-Luc2 xenograft tumor; the Balb/c nude mice bearing the tumor were administered with 50 μM of **Pt-Luc** (200 μL per 20 g mouse body weight). (b) Time-dependent bioluminescent signal generation of **Pt-Luc** from A549-Luc2 cells treated with 50 μM of **Pt-Luc**. To align with in vivo time points, bioluminescence from live cells was imaged in segments with 1-minute delays.

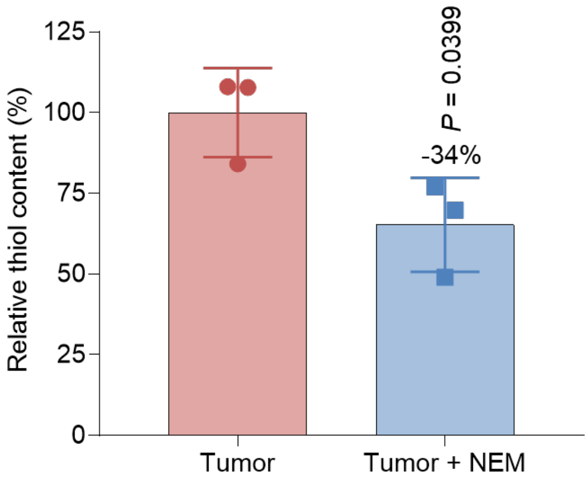

Figure S32.Thiol concentrations were measured in tumors collected from Balb/c nude mice bearing A549-Luc2 tumor xenografts, which received an intraperitoneal injection of NEM (100 μM, 200 μL per 20 g mouse body weight) or an equivalent volume of PBS for 30 minutes.

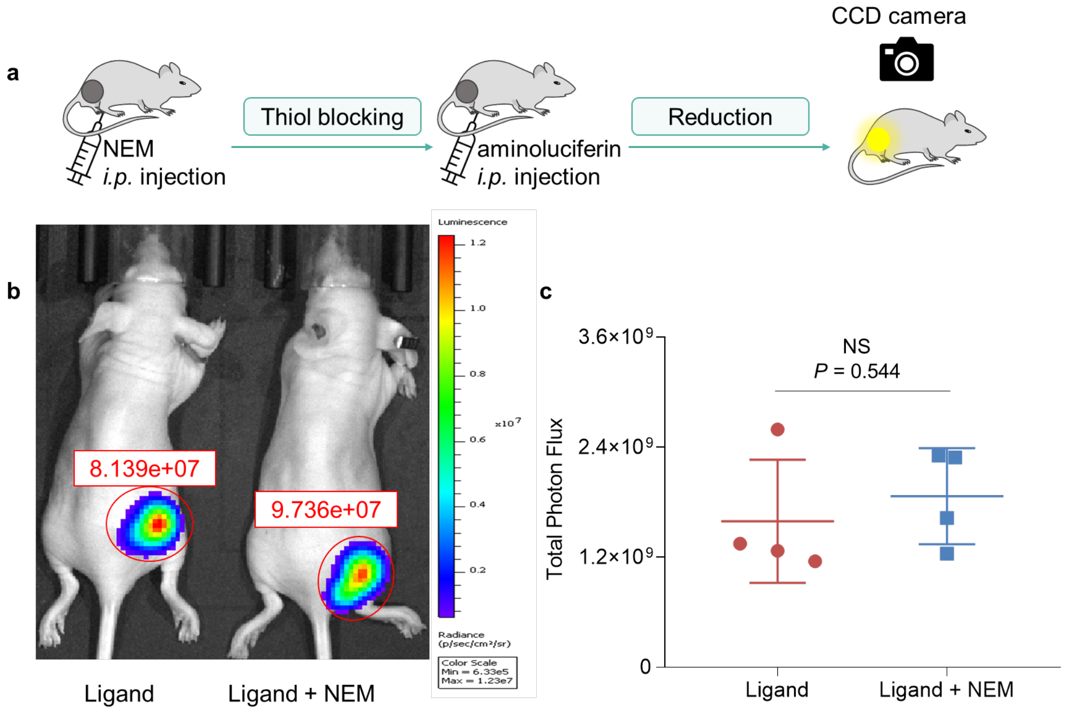

Figure S33. (a) Experimental layout for investigating the influence of thiol levels on the activity of luciferase in mice. These mice were initially administered with either vehicle or NEM, followed by aminoluciferin injection (50 μM, 200 μL per 20 g mouse body weight) 30 min later. (b) Representative images of Balb/c nude mice bearing A549-Luc2 tumor xenografts were administered with either aminoluciferin (left) or aminoluciferin with NEM (right) at 16 min. All presented experiments were repeated thrice. The bioluminescent area of the xenograft tumor, indicated by a red circle, was defined as the region of interest (ROI); the red number within the rectangle represents the total signal detected in the ROI, quantified using Living Image software 3D. (c) Total photon flux, integrated over 30 min, for cancer-bearing mice injected with aminoluciferin ± NEM. Data are presented as mean ± SD.

References

[1] H. Yao, F. Jiang, A. Lu, G. Zhang, *Int. J. Mol. Sci.* **2016**, *17*, 194.

[2] a) A. T. Aron, M. C. Heffern, Z. R. Lonergan, M. N. Vander Wal, B. R. Blank, B. Spangler, Y. Zhang, H. M. Park, A. Stahl, A. R. Renslo, E. P. Skaar, C. J. Chang, *Proc. Natl. Acad. Sci.* **2017**, *114*, 12669-12674; b) G. C. Van de Bittner, E. A. Dubikovskaya, C. R. Bertozzi, C. J. Chang, *Proc. Natl. Acad. Sci.* **2010**, *107*, 21316-21321.

[3] M. C. Heffern, H. M. Park, H. Y. Au-Yeung, G. C. Van de Bittner, C. M. Ackerman, A. Stahl, C. J. Chang, *Proc. Natl. Acad. Sci.* **2016**, *113*, 14219-14224.
[4] a) H. P. Broquist, *Nutr. Rev.* **1992**, *50*, 110-111; b) H. Yao, G. Zhu, *Dalton Trans.* **2022**, *51*, 5394-5398; c) J. X. Ong, C. S. Q. Lim, H. V. Le, W. H. Ang, *Angew. Chem. Int. Ed.* **2019**, *58*, 164-167.
[5] M. Sato, R. Kusumi, S. Hamashima, S. Kobayashi, S. Sasaki, Y. Komiyama, T. Izumikawa, M. Conrad, S. Bannai, H. Sato, *Sci. Rep.* **2018**, *8*, 968.
[6] K. F. Wallis, L. C. Morehead, J. T. Bird, S. D. Byrum, I. R. Miousse, *Environ. Mol. Mutag.* **2021**, *62*, 216-226.
[7] a) R. E. Hansen, D. Roth, J. R. Winther, *Proc. Natl. Acad. Sci.* **2009**, *106*, 422-427; b) M. Zhang, L. Wang, Y. Zhao, F. Wang, J. Wu, G. Liang, *Anal. Chem.* **2018**, *90*, 4951-4954.
[8] a) W. Chen, Y. Zhao, T. Seefeldt, X. Guan, *J. Pharm. Biomed. Anal.* **2008**, *48*, 1375-1380; b) X. Guan, B. Hoffman, C. Dwivedi, D. P. Matthees, *J. Pharm. Biomed. Anal.* **2003**, *31*, 251-261.
[9] R. Requejo, T. R. Hurd, N. J. Costa, M. P. Murphy, *FEBS J.* **2010**, *277*, 1465-1480.
[10] a) T. R. Hurd, T. A. Prime, M. E. Harbour, K. S. Lilley, M. P. Murphy, *J. Biol. Chem.* **2007**, *282*, 22040-22051; b) X.-M. Jiang, M. Fitzgerald, C. M. Grant, P. J. Hogg, *J. Biol. Chem.* **1999**, *274*, 2416-2423; c) S. M. Beer, E. R. Taylor, S. E. Brown, C. C. Dahm, N. J. Costa, M. J. Runswick, M. P. Murphy, *J. Biol. Chem.* **2004**, *279*, 47939-47951.

Author Contributions

Shu Chen leads the experimental work, data acquisition and analysis, investigation, validation, and writing of the original draft.
Wang Peng, Houzong Yao, Zhiqin Deng, Zhao Yue, Gongyuan Liu, Jiaqian Xu, Naixin Lin, Weikang Xu, and Jianbo Yue support the experimental work.
Prof. Dr. Guangyu Zhu designs the project and leads the data curation, funding acquisition, formal analysis, investigation, project administration, and validation.
